# Optimisation of measurement and data analysis of time-resolved X-ray absorption spectroscopy of thin film organic semiconductors

**DOI:** 10.1039/d6fd00089d

**Published:** 2026-09-30

**Authors:** Belen Alonso-Garcia, Miguel M. Manresa-Nalda, Oliver G. Alexander, Kaiyu Hu, Felix S. Egun, Zachery Donnellan, Joseph J. Broughton, Jacob P. Lee, David J. Hoffman, Kristjan Kunnus, Matt Bain, Douglas Garratt, Oliver Gessner, Mary R. Matthews, Jon P. Marangos

**Affiliations:** a Blackett Laboratory Prince Consort Road London SW7 2BW UK m.matthews@imperial.ac.uk; b SLAC National Accelerator Laboratory Menlo Park CA USA; c Chemical Sciences Division, Lawrence Berkeley National Laboratory Berkeley California 94720 USA; d Department of Chemistry, University of California Berkeley California 94720 USA

## Abstract

Time-resolved X-ray absorption spectroscopy (TR-XAS) at X-ray free-electron lasers enables element-specific probing of ultrafast dynamics, but soft X-ray measurements are often limited by low fluorescence yield, strong shot-to-shot fluctuations, and radiation damage in organic thin films. Here, we present a unified framework combining analytical signal-to-noise ratio (SNR) modelling with regression-based analysis for fluorescence-detected soft X-ray absorption spectroscopy. The SNR model predicts fundamentally different optimal conditions for static and transient measurements, with transient experiments requiring thin films (∼80–100 nm) and substantially higher photon counts. We apply this framework to shot-resolved total fluorescence yield XAS measurements of the non-fullerene acceptor Y6 at the nitrogen K-edge using a high-repetition-rate XFEL source, comparing low-dose and intermediate-dose regimes relevant to transient and static measurements, respectively. A calibration-based flux reconstruction systematically improves the fluorescence–flux correlation, while the dominant improvement arises from reformulating XAS extraction as a shot-resolved regression problem. Compared to the conventional ratio-of-means estimator, the regression-based estimator reduces the run-to-run spectral variability by up to a factor of ∼4.4 in the low-dose regime most relevant for transient measurements. Residual-based outlier rejection was found to systematically degrade reproducibility, indicating that the tails of the shot distribution primarily reflect intrinsic XFEL fluctuations rather than detector artefacts. These results demonstrate that reliable soft X-ray TR-XAS requires the joint optimisation of experimental design and statistical inference, providing a practical framework for extracting robust observables from low-signal XFEL measurements in dose-limited regimes.

## Abstract 2

Combining dose-aware experimental design with shot-resolved regression enables reliable fluorescence-detected XAS in soft X-ray XFEL experiments on dose-limited samples, reducing run-to-run spectral variability by up to 4.4-fold.

## Graphical abstract

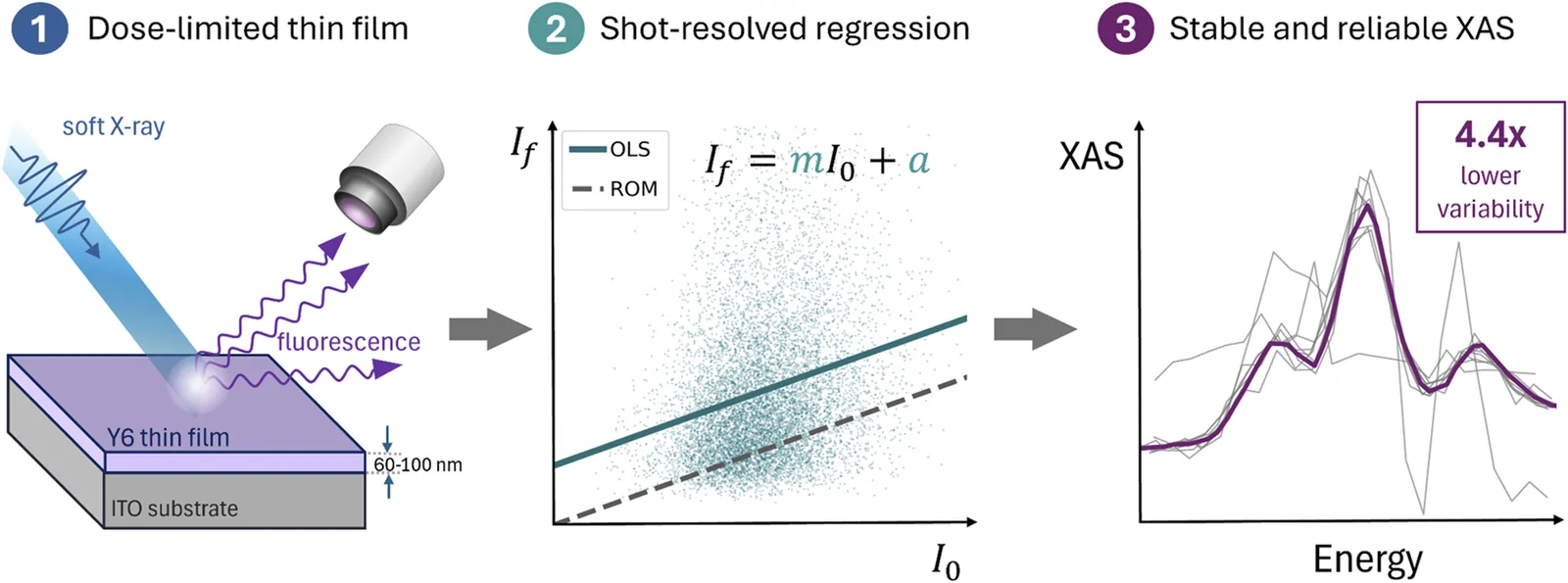

## 1 Introduction

X-ray absorption spectroscopy (XAS) provides unprecedented capabilities for investigating molecular electronic structure. The high photon energy of X-rays enables access to core-level electronic orbitals, which are localised at an atomic site and are distinct for each element. This element-specificity allows the probing of particular atomic sites within molecules, removing undesired contributions.^1,2^ In particular, Near Edge X-ray Absorption Fine Structure (NEXAFS) is dependent on the unoccupied density of states.^3,4^ Thus, being sensitive to the atom’s oxidation state, it provides a direct probe of the local charge density at the target atom.^2,5^

To extend these capabilities into the ultrafast regime, advanced X-ray sources are required. The development of ultrafast X-ray sources has expanded these static capabilities into a time-resolved tool for tracking the temporal evolution of photo-initiated molecular dynamics.^6–8^ Table-top high harmonic generation (HHG) sources producing attosecond to femtosecond pulses with high temporal coherence have pioneered these studies.^9,10^ However, a low photon flux within the “water window”^11^ (∼280–540 eV) and the inaccessibility of higher energy edges remain their main limitations. This is a consequence of the rapidly decreasing conversion efficiency of the HHG process which occurs as the wavelength of the driving laser is increased, a necessary step to generate higher energy photons (above 250 eV).^12–14^ In contrast, X-ray free electron lasers (XFELs) achieve photon fluxes orders of magnitude higher than HHG sources at photon energies across the X-ray range.^15^

Significant progress has been made in increasing repetition rates, with LCLS-II designed to reach up to 1 MHz^16^ and the European XFEL up to 27 kHz in burst mode (4.5 MHz intra-train, 10 Hz inter-train).^17^ These high repetition rates provide the high-count statistics required for photon-hungry spectroscopies and measurements on dilute samples.^18–21^ However, the stochastic nature of the main operation mode, self-amplified spontaneous emission (SASE), causes significant shot-to-shot intensity and spectral variations.^22–25^ Consequently, intensity normalisation at the single-shot level becomes essential. Additionally, the challenging synchronisation of XFELs with optical pump pulses results in a timing jitter, also requiring per-shot estimation of the pump–probe delay.^26,27^ Despite these experimental challenges, the high photon flux and temporal resolution of XFELs make them an essential tool for time-resolved XAS across diverse photophysical and photochemical systems.^28–30^

A key class of systems where such capabilities are particularly valuable are organic semiconductors, which underpin organic photovoltaic (OPV) devices and aim to transform solar energy harvesting. They promise lightweight, non-toxic, and flexible devices compared to inorganic solar cells.^31–33^ However, their power conversion efficiencies (PCEs) remain modest, in part due to the low dielectric constant of organic materials^34^ (*ε*_r_ ≈ 2–4), compared to inorganic semiconductors (*ε*_r_ ≈ 11–12). This causes excitons to be strongly bound, with binding energies on the order of hundreds of meV, which hinders the generation of free charge.^35–37^ The shift from fullerene to non-fullerene acceptors (NFAs) has resulted in substantial PCE increases, particularly for donor-polymer blends using Y6 or its derivatives as the acceptor.^38–40^ The molecular structure of Y6 (Fig. 1(a)) consists of alternating donor (D) and acceptor (A) units in an A–DA′D–A configuration, which facilitates intramolecular charge transfer.^41^ However, the charge generation mechanism is not yet well understood, with the nature of the intermediate states still actively debated.^42–44^ Leveraging the element-specificity and sensitivity to the local charge environment of XAS offers a powerful route to directly interrogate the charge dynamics of OPVs,^45^ including non-fullerene acceptors.

**Fig. 1:**
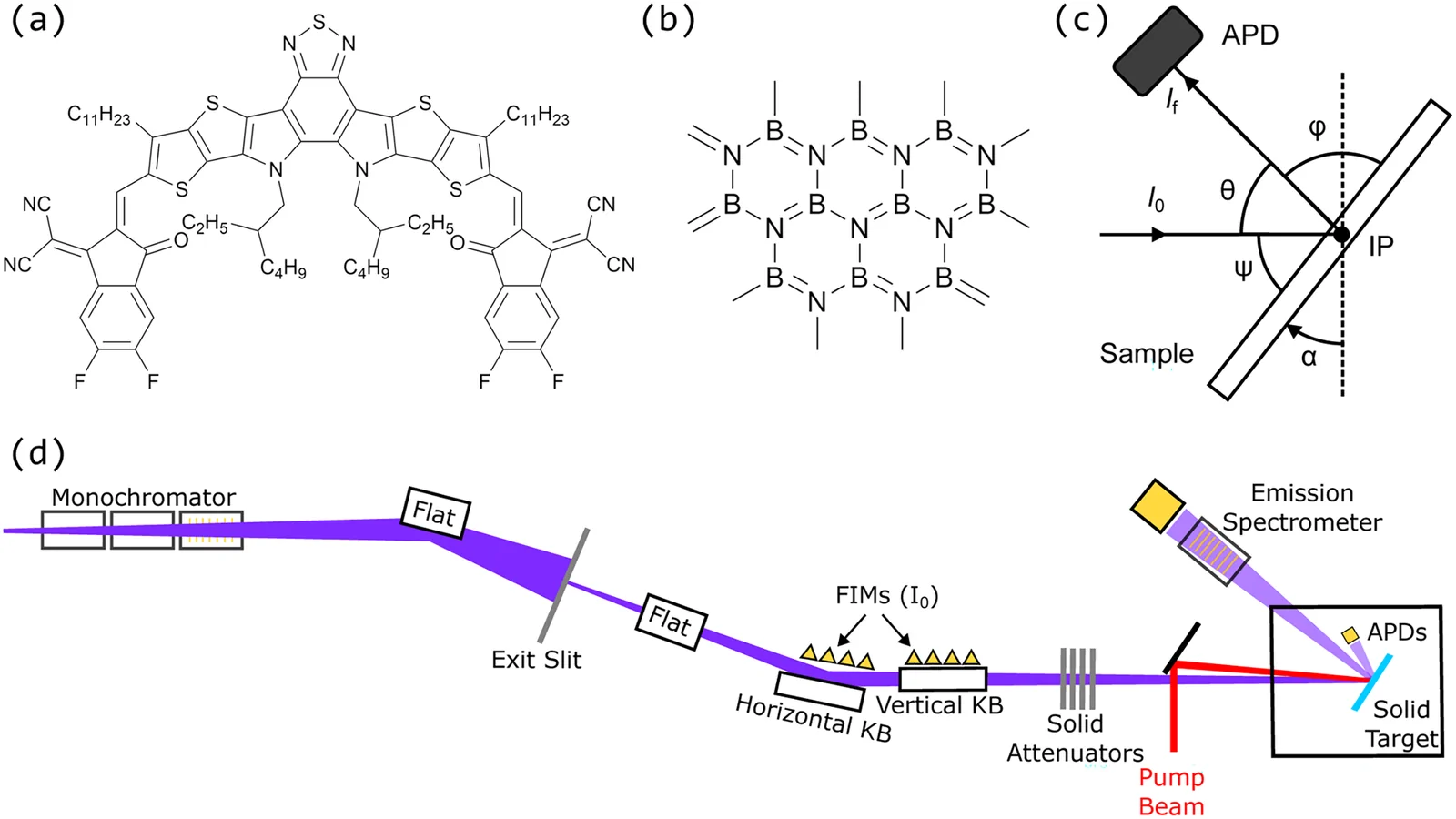
(a and b) Chemical structures of the samples studied: Y6 and hexagonal boron nitride (hBN). (c) Detection geometry, where *θ* is the sample rotation angle, *ψ* the incident angle relative to the sample surface, and *ϕ* the fluorescence emission angle. IP denotes the interaction point. (d) Simplified schematic of the chemRIXS endstation at LCLS-II showing the components relevant to this experiment (adapted from ref. 21 under the terms of the Creative Commons Attribution 4.0 International (CC BY 4.0) License, copyright 2026, D. Hoffman).

The application of XAS to OPVs poses challenges that are applicable to other low bandgap condensed phase materials. A primary challenge is the combination of the low radiation damage threshold of organic thin films with the high flux delivered by XFELs,^46^ requiring mitigation strategies such as beam attenuation or rapid sample scanning.^47,48^ Furthermore, as they consist of lighter elements, non-radiative Auger decay dominates, and the fluorescence yield is low.^49^ The absorption cross-section of the element of interest can also be small relative to the total absorption background,^50^ which is further obscured by detector noise. As a result, these measurements are inherently limited by a low signal-to-noise ratio (SNR).^4,46^ In addition, shot-to-shot normalisation is required to correct for intrinsic SASE intensity fluctuations.^51^

Although unambiguous measurements of ultrafast charge dynamics are challenging, the interpretation of both static and transient spectra additionally relies on accurate modelling of the underlying electronic structure and dynamics. Inaccurate processing or low signal quality can introduce artefacts that obscure the physical interpretation. It is therefore essential to maximise the experimental SNR and develop robust methods for extracting reliable observables, particularly in systems exhibiting delocalised excitations.

In this work, we address these challenges through a combination of predictive modelling and data-driven analysis. First, we develop an analytical model for the SNR in both transmission and fluorescence XAS, extending previous frameworks^46,52^ to account for thin-film geometries and self-absorption effects. Second, we introduce a regression-based framework for extracting XAS from shot-resolved fluorescence data under conditions where conventional normalisation fails. The model and optimization procedure is benchmarked with XAS measurements of the NFA Y6 at the nitrogen K-edge at 5.5 kHz using the LCLS-II free electron laser. Together, these approaches show that reliable TR-XAS measurements require optimised design, implementation, and analysis, providing a general framework for both maximising signal quality and extracting robust observables.

## 2 Detection schemes and experimental challenges in soft X-ray TR-XAS

The extraction of XAS from XFEL measurements depends critically on the detection scheme. In transmission-mode XAS, the absorption coefficient is obtained directly from the Beer–Lambert relation,1
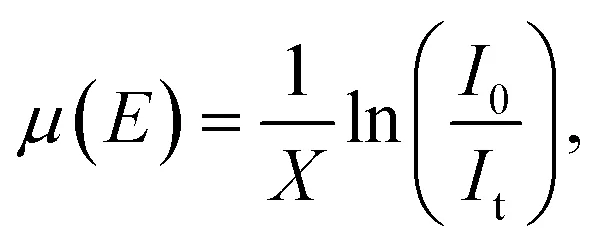
where *I*_0_ and *I*_t_ denote the incident and transmitted intensities, respectively, and *X* the interaction path length. Transmission detection provides a direct and quantitative measure of the absorption coefficient and is free from fluorescence self-absorption effects. At XFELs, both signals can be recorded on a shot-by-shot basis, enabling robust pulse-resolved normalisation. However, this geometry requires samples of well-defined thickness and sufficient transmission, which can be difficult to realise for thin films on substrates.

Fluorescence-based detection methods are therefore widely employed in soft X-ray experiments. In total fluorescence yield (TFY), the absorption spectrum is inferred from the emitted fluorescence intensity as a function of incident photon energy. In the dilute or thin-sample limit, the detected fluorescence intensity is approximately proportional to the absorption coefficient, TFY(*E*) ∝ *µ*(*E*), since the number of emitted photons scales with the number of core-level excitations.

Despite its simplicity, TFY is subject to significant limitations. In concentrated or thick samples, two distinct distortion mechanisms arise: saturation of the incident beam and self-absorption of emitted fluorescence photons. Although these can partially compensate one another in thick solid-state samples,^53^ both generally cause the proportionality between TFY and *µ*(*E*) to break down, introducing nonlinearities.^54^ In such cases, the measured TFY signal reflects a convolution of absorption and attenuation processes,^55^ and significant spectral distortions can arise (exceeding 50% in extreme cases^56^). Additionally, for systems with strong multiplet character, such as transition metals, the fluorescence yield depends on the final state, causing TFY to deviate from the true absorption cross-section.^55^ In the soft X-ray regime, background fluorescence from light elements further limits the SNR, particularly in organic materials.^57^

More advanced photon-in/photon-out techniques provide improved spectral selectivity. In resonant inelastic X-ray scattering (RIXS), both the incident and emitted photon energies are resolved, yielding detailed information on the participating electronic excitations and occupied valence orbitals. Partial fluorescence yield (PFY) can be obtained by integrating the emission over a selected energy window, allowing for background suppression and enhanced element selectivity.^3,58,59^ By selecting specific final states, PFY can reduce multiplet-induced distortions and improve spectral fidelity.^21,54^

For thin-film OPV systems, these detection challenges are further compounded by the interplay between sample thickness, excitation fraction, and fluorescence yield. Increasing the sample thickness enhances fluorescence signal levels but reduces the fraction of photoexcited molecules probed by the X-ray pulse, while thinner samples improve excitation at the expense of signal intensity. This trade-off, combined with the limited radiation tolerance of organic materials, makes the optimisation of experimental conditions non-trivial.

These considerations show that the measured fluorescence signal reflects a coupled dependence on absorption, emission, and geometry. As a result, the achievable SNR cannot be optimised by simple intuitive arguments. In the following section, we develop a quantitative model to describe these dependencies and predict the achievable SNR under realistic experimental conditions.

## 3 Theoretical signal-to-noise model for thin-film TR-XAS

The considerations outlined above highlight that the achievable SNR in TR-XAS is governed by the interplay between absorption, detection geometry, and photon statistics. General frameworks for SNR in X-ray absorption spectroscopy have been established for static measurements^52^ and extended to time-resolved transmission geometries.^46^

Here, we extend these approaches to thin-film systems and fluorescence detection geometries relevant to TFY and PFY measurements. The model incorporates sample thickness, experimental geometry, and excitation fraction, providing a unified description of the SNR for static and pump–probe XAS.

The framework can also be used prospectively to guide experimental design. For a specified target SNR, the model can be inverted to estimate the required incident photon count and, for a known photon flux and repetition rate, the corresponding acquisition time. Application to different samples, absorption edges, or excitation conditions requires only the relevant material and experimental parameters, such as absorption coefficients, fluorescence yield, sample density, detection geometry, and optical excitation conditions. These quantities can generally be obtained from tabulated databases or independent measurements. The model therefore provides a general means of assessing measurement feasibility and the photon statistics required to reach a target SNR, subject to practical limitations such as detector noise, XFEL fluctuations, and radiation damage.

### 3.1 General framework

We consider a uniform sample of thickness *X* consisting of the element of interest *A*, whose absorption edge is probed, and all other elements present, collectively denoted as *B*. The total absorption coefficient of the sample at photon energy *E* is written as

2 *µ*_s_(*E*) = *µ*_*A*_(*E*) + *µ*_*B*_(*E*),

where3
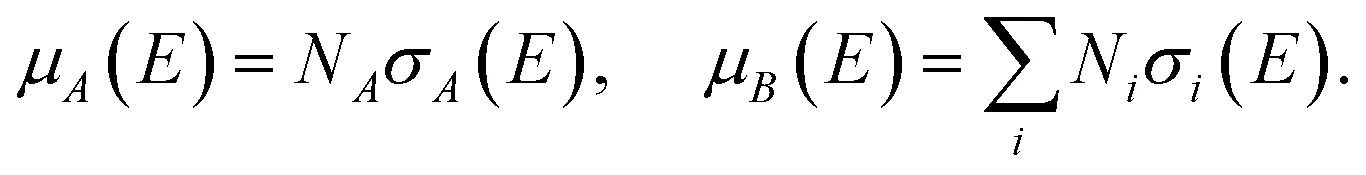
Here *N*_*i*_ is the number density of atomic species *i* and *σ*_*i*_(*E*) is the corresponding atomic absorption cross-section.

The XAS signal arises from a change in the absorption coefficient of the element of interest, Δ*µ*_*A*_, corresponding to either the static contrast across an absorption edge or a pump-induced change in a time-resolved experiment.

### 3.2 Fluorescence detection

We first consider fluorescence-based detection, which is the most commonly employed geometry for thin-film soft X-ray measurements. In this case, the measured signal is determined not only by the absorption of the incident X-rays, but also by the attenuation of the emitted fluorescence photons within the sample.

For a sample of thickness *X*, incident angle *ψ*, and emission angle *ϕ*, the fluorescence intensity is given by^52^4
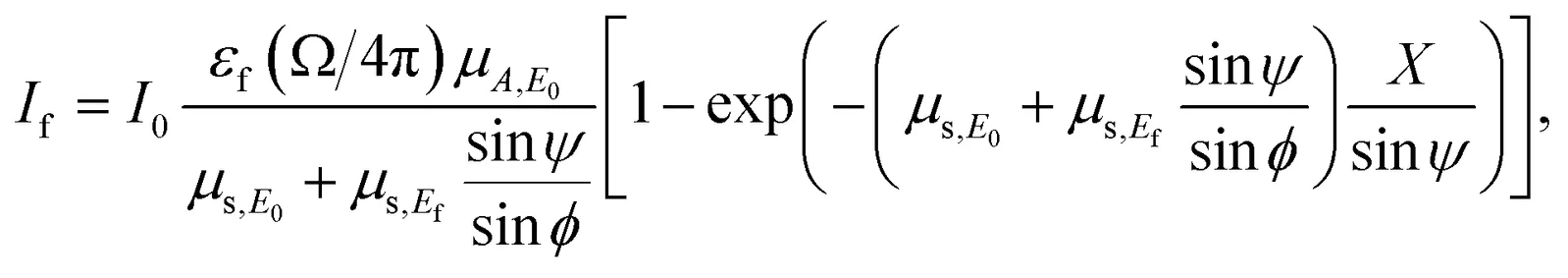
where *I*_0_ is the incident intensity, *ε*_f_ is the fluorescence yield, Ω is the detector solid angle, and *E*_0_ and *E*_f_ are the incident and fluorescence photon energies, respectively.

For compactness, we define

5 *C* = *ε*_f_(Ω/4π),

6
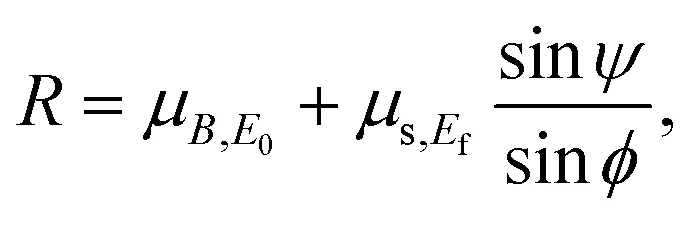
7
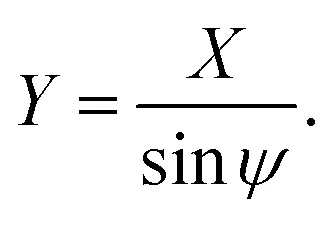


#### 3.2.1 Static XAS

In static XAS, the signal is defined as the change in the fluorescence response due to a variation in the absorption coefficient of the element of interest:8
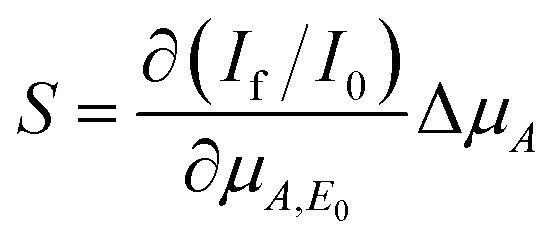
9
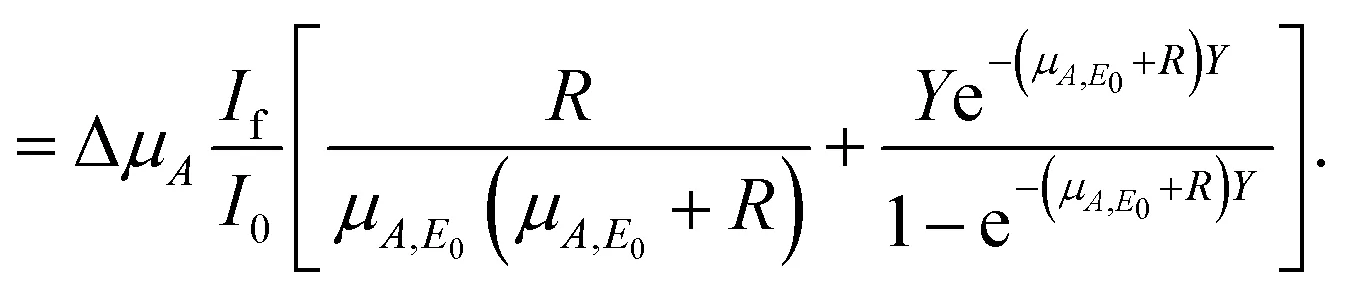


Assuming shot-noise-limited detection and neglecting fluctuations in *I*_0_, the noise is given by10
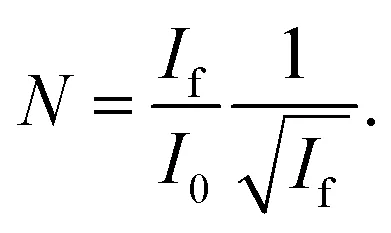


The resulting SNR is therefore11
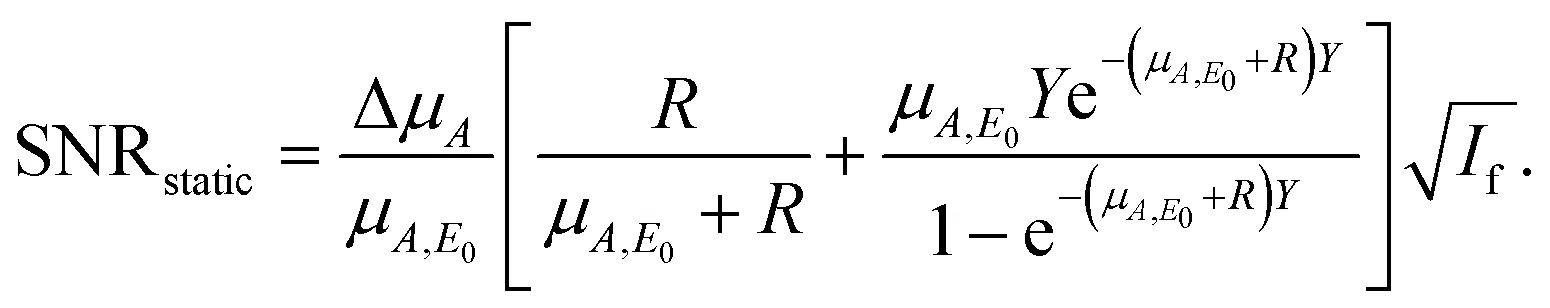


The thickness dependence of the SNR is therefore governed by12
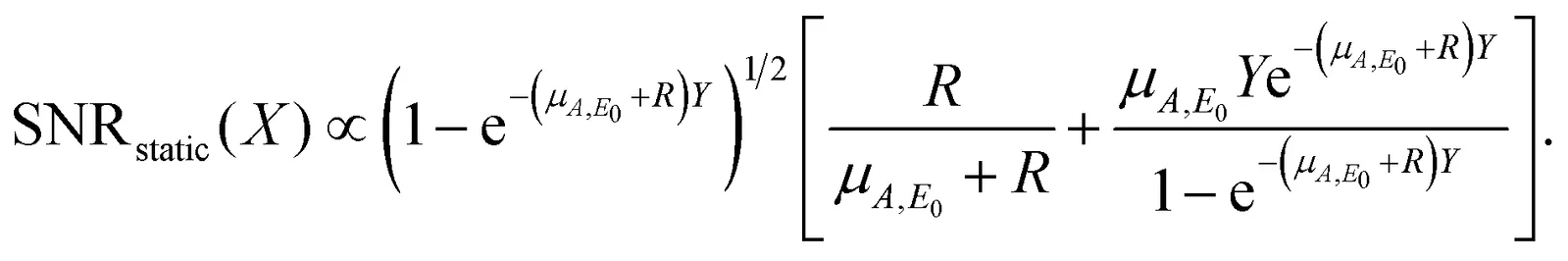


In the thin-sample limit, (*µ*_*A*,*E*_0__ + *R*)*Y* ≪ 1, the fluorescence intensity scales linearly with thickness and the SNR behaves as 
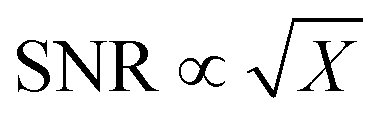
. In contrast, in the thick-sample limit, the fluorescence signal saturates, leading to a corresponding saturation of the SNR.

#### 3.2.2 Pump–probe XAS

In a time-resolved experiment, the optical pump induces a change in the absorption coefficient, Δ*µ*_p_ = *η*Δ*µ*_*A*_, where *η* is the excitation fraction. For a pump fluence *F*_p_, wavelength *λ*, and optical absorption coefficient *µ*_*λ*_, the excitation fraction is given by^46^13
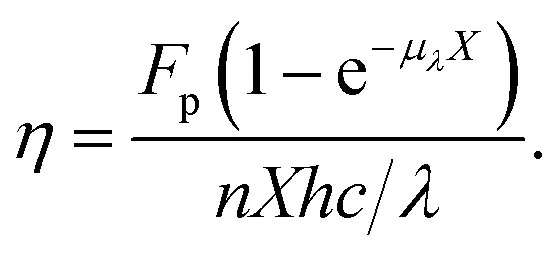


Following an expansion in the small-pump limit (*µ*_p_ ≪ *µ*_*A*,*E*_0__), the pump–probe SNR becomes14
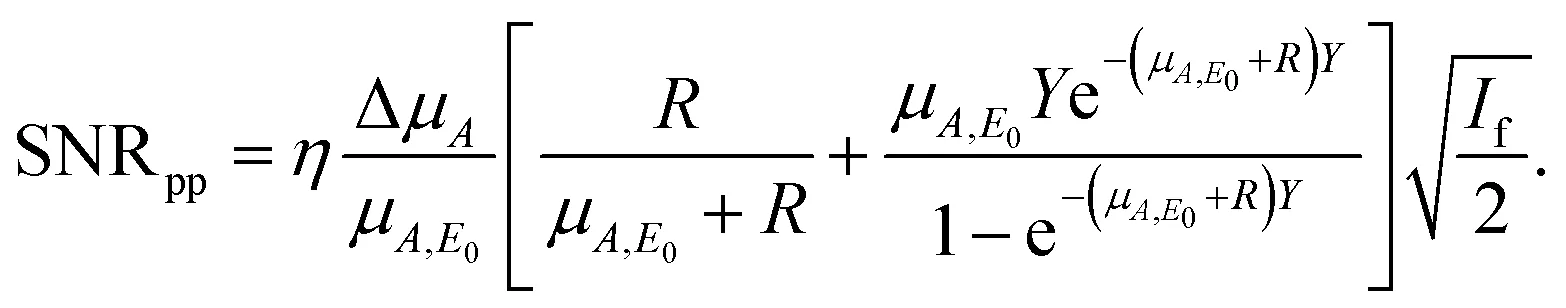


Comparing with eqn (11), we obtain the general relation15
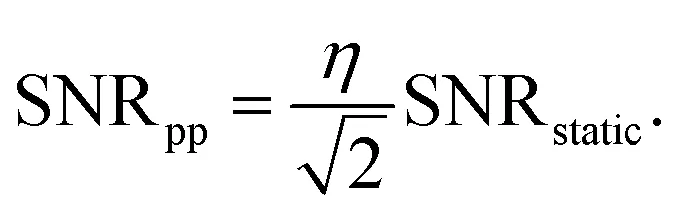


This result shows that the pump–probe SNR can be directly related to the static SNR, reduced by the excitation fraction and an additional factor of 
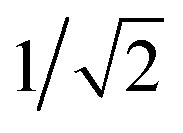
 arising from the subtraction of pumped and unpumped signals.

### 3.3 Transmission detection

For completeness, we briefly summarise the corresponding expressions for transmission detection. In the static case, the SNR is given by^52^16
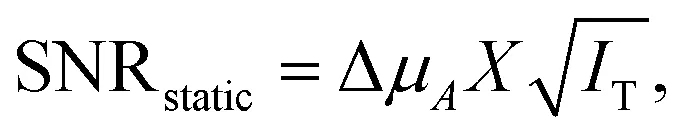
with a thickness dependence

17 SNR(*X*) ∝ *X*e^−*µ*_s_*X*/2^.

In the pump–probe case, the SNR reduces in the small-signal limit to^46^18
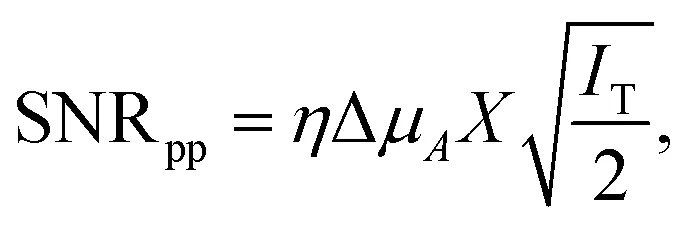
which follows the same general relation19
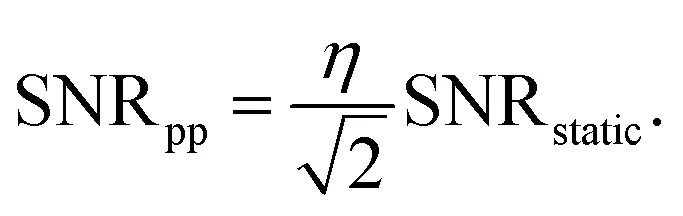


Thus, for both fluorescence and transmission detection, the static SNR provides a direct route to estimating the pump–probe SNR.

While this model captures the fundamental limits imposed by photon statistics and geometry, the practical extraction of XAS from fluorescence measurements at XFELs is further complicated by shot-to-shot fluctuations and imperfect flux normalisation. In particular, under conditions of low fluorescence yield, the correlation between the measured incident (*I*_0_) and emitted (*I*_f_) signals can be significantly degraded. This motivates the development of shot-resolved analysis frameworks for extracting XAS in regimes where conventional normalisation approaches become unreliable, which we address in the following section.

## 4 Extraction of XAS from total fluorescence yield

In practice, the extraction of XAS from fluorescence measurements is not uniquely defined, but depends on how the relationship between incident and emitted signals is statistically inferred. This is particularly important in XFEL measurements, where shot-to-shot fluctuations and low fluorescence yield degrade the correlation between signals. We therefore examine different estimators for extracting the XAS observable from shot-resolved data.

### 4.1 Measuring TFY-XAS

At XFELs, TFY-XAS spectra can be obtained by scanning incident photon energy using a monochromator. For each nominal energy point, data is acquired over many XFEL shots and subsequently grouped into energy bins to account for residual shot-to-shot energy jitter. Both the incident and fluorescence signals are measured on a shot-resolved basis. For the *j*th shot, these can be denoted as *I*_0,*j*_ and *I*_f,*j*_, respectively.

The incident and emitted pulse energy are typically measured with avalanche photodiodes (APDs), microchannel plate detectors (MCPs) or a combination of both, like fluorescence intensity monitors (FIMs).^60,61^ For each XFEL shot, the detector outputs are digitised, with the recorded waveforms consisting of a transient positive peak corresponding to the detected photon signal, followed by a recovery tail. The signal amplitude for that shot is obtained by integrating the waveform over a predefined time window.

In the ideal case, the fluorescence signal measured is proportional to the incident flux on a shot-to-shot basis, such that

20 *I*_f,*j*_ ∝ *I*_0,*j*_.

The constant of proportionality is in turn proportional to the fluorescence intensity given by eqn (4). An additional factor is introduced by the electronics of the detector and although this factor is unique to the experimental setup and difficult to determine *a priori*, it is constant and can be calibrated using a known sample if needed. We can combine all prefactors other than the absorption coefficient into a constant *G* and express this relationship as

21 *I*_f,*j*_ = *Gµ*_*A*_*I*_0,*j*_.

Under this assumption, an estimate of the TFY signal at a given energy, *μ̂*_*A*_, is obtained using the ratio-of-means (ROM) estimator,22
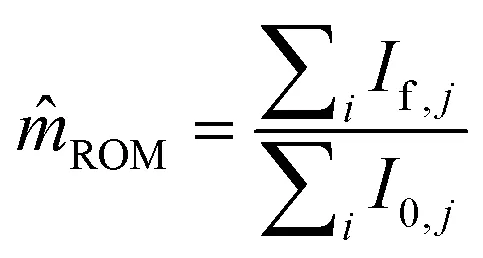
23
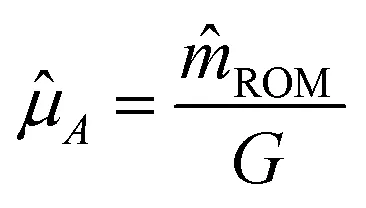


The reason this cannot be extracted from a single shot is that this ignores the statistical properties of the fluorescence and any detector noise. In low-yield regimes, such as with thin organic films, the fluorescence signal can approach the detector noise floor and the counting statistics of signal detection become very important. A noise term *ξ*_*i*_ must be added and instead of being well defined, the fluorescence intensity at a given incident intensity is sampled from a Poisson distribution, Pois, with a well-defined mean:

24 *I*_f,*j*_ ∼ Pois(*Gµ*_*A*_*I*_0,*j*_) + *ξ*_*i*_.

*I*_0,*j*_ is also sampled from a random distribution, which for a monochromated SASE beam is typically also a Poisson distribution, *i.e.*,25
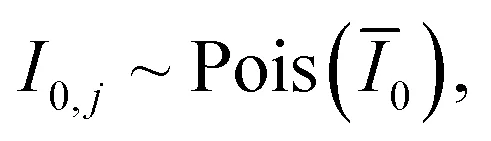
with 
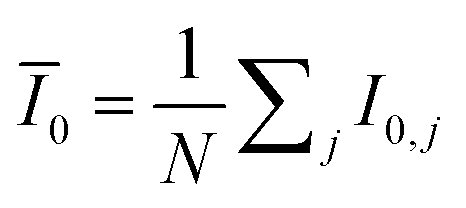
.

The TFY signal at a given energy is then the maximum likelihood estimator of *µ*_*A*_. For an unbiased noise term and in this ideal Poisson–Poisson case, this is still given by eqn (22) and (23); however, as we show in Section 5.2.3, this is not always the best estimator with real data.

### 4.2 Least-squares linear fitting approach

An intuitive alternate estimator for extracting the XAS signal from shot-resolved data is to consider a least-squares fit (linear regression) between the fluorescence and incident monitors. Defining the ordinary least squares (OLS) slope and intercept as *m̂*_OLS_ and *â*_OLS_26
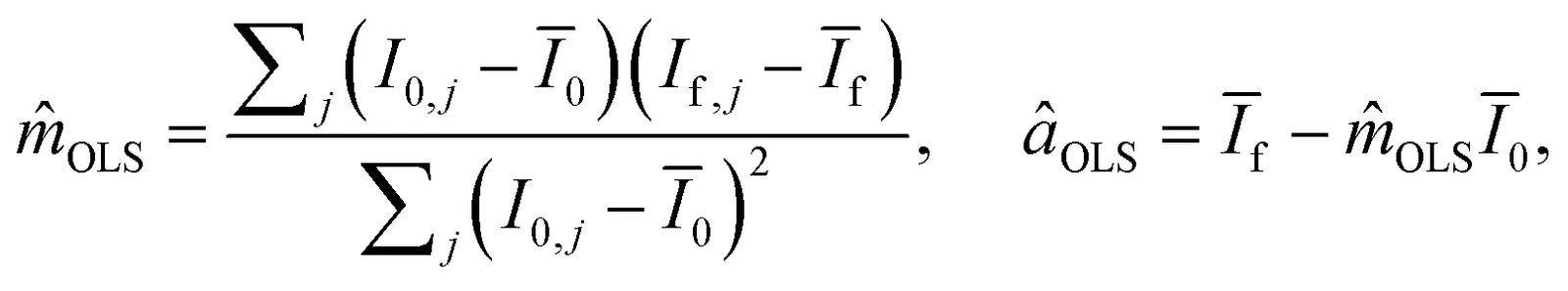


In the ideal Poisson–Poisson model of eqn (22), the intercept is expected to be zero. In practice, however, including *a* can be important if there are detector offsets or sources of background noise.

It is useful to note that *m̂*_ROM_ is itself a weighted least-squares solution (WLS) objective for a through-origin fit,27
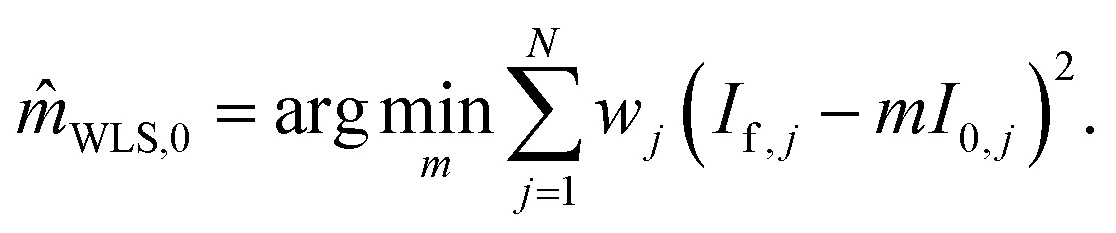
The minimiser is28
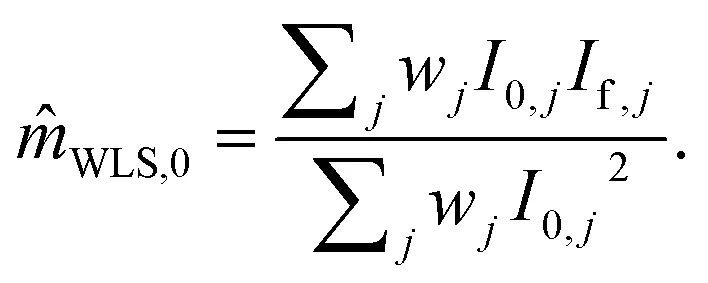
Therefore, with weights *w*_*j*_ = 1/*I*_0,*j*_,29
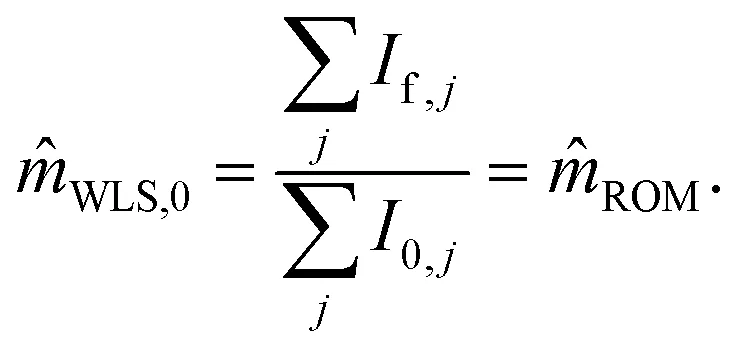
Thus, the ROM estimator corresponds to a through-origin WLS regression with weights inversely proportional to the measured incident flux. As such, the ROM approach places a greater importance on shots with low incident energy and fluorescence yield than an OLS approach. This is not optimal for the ideal Poisson–Poisson case (eqn (24) and (25)); however, where there are deviations from this ideal case that are difficult to characterise, as is frequently the case with XFELs, it is logical to take an empirical approach, using the method which leads to the greatest reproducibility in repeat measurements.

Common sources of error which lead to deviation from a Poisson distributed fluorescence are detector dark counts, stray light, and readout noise. A regression that includes an intercept can in some cases absorb these effects into *â* and place greater weight on data points with high flux, where noise levels are relatively small.

Furthermore, these estimators treat *I*_0,*j*_ as known exactly. In practice, the incident monitor itself is noisy. Errors in *I*_0,*j*_ lead to the errors-in-variables problem, causing regression dilution and the systematic underestimation of the absorption. In some cases outlier rejection may be warranted, again using the reproducibility results in repeat measurements as verification.

Although we are advocating for an empirical approach to selecting the best estimator for a dataset, we focus on an objective measure in the repeatability of measurements, which is independent of the measurement result itself, and caution against choosing an estimator based on the derived result itself. This would even apply in a case where a ground truth is known; for example, choosing a method that gives a result that is closest to a reference spectrum is inadvisable as this biases the outcome of the measurement. In the absence of repeat measurements, objective measurements based on data resampling similar to *k*-folds cross validation could be used.

### 4.3 Calibration-based flux reconstruction

A central requirement for reliable TFY-XAS extraction is an accurate and unbiased estimate of the incident flux on each shot, *I*_0,*j*_. Even small amounts of nonlinearity, common in APDs and MCPs, can have a significant adverse effect on results and lead to a poor correlation between measures of *I*_0_ and *I*_f_. Fortunately, general methods can be used to characterise and correct nonlinearity in detectors.

In this work, we employ a calibration-based approach^60^ to construct an improved proxy for the incident flux. The method uses a reference sample with strong and well-behaved fluorescence response (hexagonal boron nitride, hBN, Fig. 1(b)), for which the proportionality between fluorescence and incident flux is well satisfied.

For each shot *j*, we denote the set of signals from the incident flux detectors as a vector 
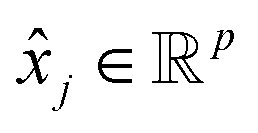
, where *p* = *N*_traces_ × 4 corresponds to the number of digitised time traces and detector channels. These signals provide a high-dimensional measurement of the incident X-ray pulse. We then construct a through-origin linear regression model that maps these signals onto a scalar fluorescence response for the reference sample

30 *I*^(pred)^_f,*j*_ = *m̂*·*x̂j*,

The coefficients *m* = (*m*_1_, …, *m*_*p*_) are determined using the 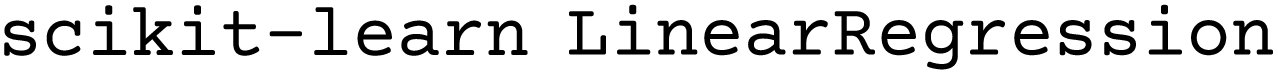 algorithm by minimising the residual sum of squares between the observed *I*^(ref)^_f,*j*_ reference dataset and the predicted *I*^(pred)^_f,*j*_31
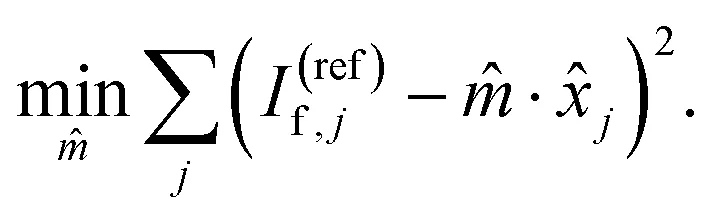


The resulting model provides a prediction of the fluorescence signal that would be observed from the reference sample for a given XFEL shot. Since, for the reference, *I*^(ref)^_f_ ∝ *I*_0_, the predicted quantity *I*^(pred)^_f,*j*_ can be interpreted as a calibrated proxy for the incident flux,

32 *I*^(pred)^_0,*j*_ ∝ *I*^(pred)^_f,*j*_.

In the following, we therefore use *I*^(pred)^_0,*j*_ as an alternative estimate of the incident flux. This quantity incorporates information from all available detector channels and implicitly corrects for detector nonlinearities and geometric effects learned during calibration.

The TFY signal for the target sample can then be estimated using either the ratio-of-means estimator (eqn (22)) or the regression-based estimator (eqn (26)), replacing *I*_0,*j*_ with *I*^(pred)^_0,*j*_. As shown in Section 5.2.3, this approach improves the correlation between fluorescence and incident signals and leads to more stable XAS extraction, particularly in low-yield regimes.

## 5 Results and discussion

All experimental results presented in this work were obtained from static TFY-XAS measurements at the nitrogen K-edge at the chemRIXS endstation of LCLS-II.^21^ A schematic of the experimental setup and detection geometry is shown in Fig. 1(c and d). Fluorescence signals were recorded on a shot-resolved basis using APDs, while the incident X-ray flux was measured independently upstream using FIMs. The analysis presented here focuses on TFY, excluding spectrally resolved PFY data obtained with the emission spectrometer.

The samples studied were thin films of the organic semiconductor Y6 deposited on indium tin oxide (ITO) substrates. The nitrogen atoms in Y6 occupy chemically distinct environments, giving rise to multiple resonances at the N K-edge,^62^ making the N K-edge well suited for probing site-specific changes in the local electronic structure. The primary datasets analysed in this work were acquired from 60 nm Y6 films using a grazing-incidence geometry with *ψ* ≈ 60°, *θ* = 60°–70°, and *ϕ* = 50°–60°. In addition, a separate dataset acquired in a different geometry (*ψ* = 90°, *ϕ* = 45°) is presented to compare the effect of sample thickness using both 60 nm and 100 nm films.

Energy calibration of the monochromator was performed using a hexagonal boron nitride (hBN) reference sample. The measured spectrum was aligned to literature values^63^ using the σ* resonance at 409 eV and its lower-energy shoulder at 406.8 eV, establishing the conversion between monochromator position and photon energy.

The XFEL operated in burst mode with an 8.3 kHz base repetition rate and a 66% duty cycle, corresponding to an effective repetition rate of 5.5 kHz. The X-ray beam size (1/*e*^2^ diameter) at the interaction point was approximately 440 × 317 µm^2^. The pulse energy at 400 eV was ∼1.4 J, corresponding to an unattenuated peak fluence of 2.56 mJ cm^−2^. The incident flux was controlled using aluminium filters of thickness 780 nm and 400 nm, yielding peak fluences of approximately 0.17 and 0.63 mJ cm^−2^, respectively.

To mitigate radiation damage, the sample was continuously rastered. The accumulated photon dose per unit area as a function of raster speed (*v*) is given by

33 *N*_*γ*_(*v*) = *N*_*γ*,pulse_ × *N*_shots_(*v*),

with34
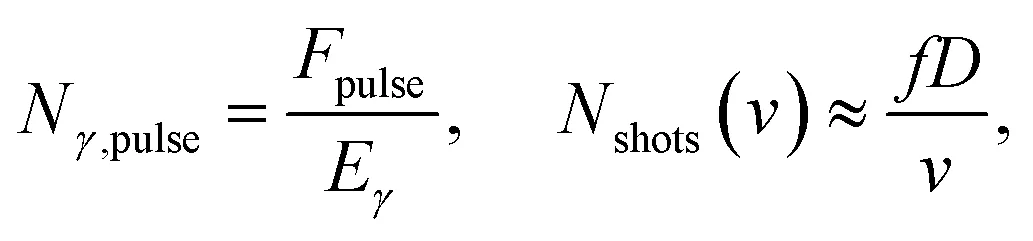
where *F*_pulse_ is the pulse fluence, *E*_*γ*_ the photon energy, *f* the repetition rate, and *D* the beam diameter along the scan direction.

For a raster speed of 300 µm s^−1^, the accumulated dose is approximately 1.5 × 10^16^ photons cm^−2^ using the 780 nm filter and 5.6 × 10^16^ photons cm^−2^ using the 400 nm filter. At doses ≲2 × 10^16^ photons cm^−2^, no measurable radiation damage was observed, although the fluorescence signal remained weak, resulting in low *I*_0_–*I*_f_ correlation and reduced spectral stability. At intermediate doses (5 × 10^16^ ≲ *N*_*γ*_ ≲ 10^17^ photons cm^−2^), stronger fluorescence signals improved correlation, although signatures of optical bleaching began to appear. At higher doses (≳10^17^ photons cm^−2^), both optical bleaching and changes in the X-ray spectra were observed.

These observations define two experimentally relevant operating regimes used throughout this work: a low-dose, dose-limited regime (*N*_*γ*_ ≲ 2 × 10^16^ photons cm^−2^), representative of transient measurements where preservation of the optical response is essential, and an intermediate-dose regime (5 × 10^16^ ≲ *N*_*γ*_ ≲ 10^17^ photons cm^−2^), where higher signal levels improve counting statistics but approach the onset of radiation-induced effects. Together, these regimes provide a controlled framework for evaluating the interplay between signal level, flux correlation, and XAS extraction methodology.

Prior to the experiment, the SNR model developed in Section 3 was used to guide the choice of experimental parameters and assess the feasibility of the measurements. The trends in signal stability, correlation, and thickness dependence presented in Section 5.2.5 are consistent with the predictions of this model.

### 5.1 SNR results for static and transient XAS

We first evaluate the SNR model under the relevant experimental conditions to compare the requirements for static and transient TFY-XAS measurements.

The calculations were performed using representative experimental and material parameters. The detection geometry was taken as *ψ* = 90° and *ϕ* = 45°, with an effective detector efficiency and solid angle acceptance of the order of 0.01 ppm. For the transient case, an 800 nm optical pump with a fluence of 300 µJ cm^−2^ was assumed, together with a Y6 optical absorption coefficient of *µ*_*λ*_ = 10.9 µm^−1^ at the pump wavelength.^46^ X-ray absorption and fluorescence emission energies were taken from ref. 64 and 65, with corresponding absorption coefficients from ref. 66.

The dependence of the SNR on sample thickness is shown in Fig. 2(a). For the transient case, the SNR exhibits a well-defined maximum, allowing the optimal thickness to be obtained directly from the SNR curve. In contrast, the static fluorescence SNR increases monotonically and approaches a plateau for *X* > 0.5 µm, making the definition of a unique optimum less straightforward.

**Fig. 2:**
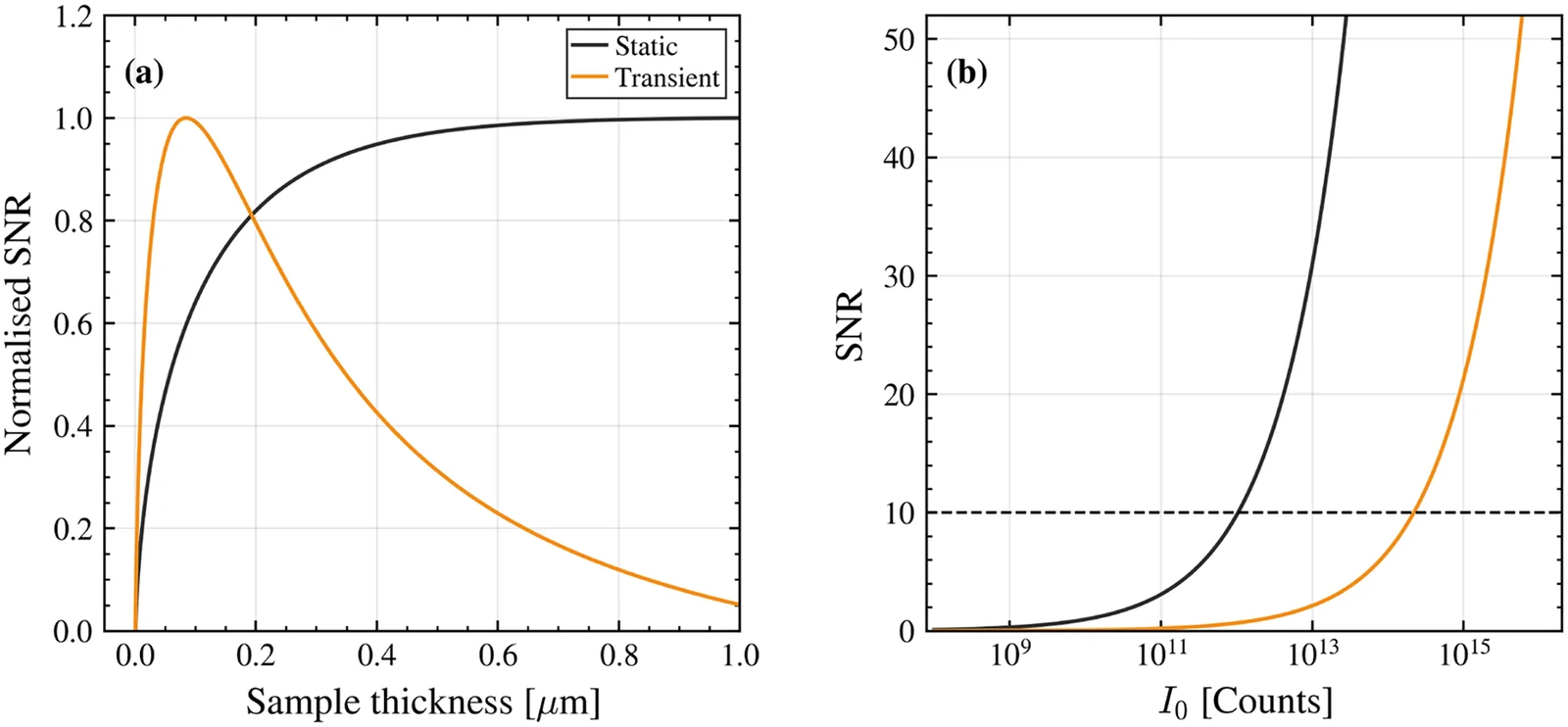
(a) Normalised SNR as a function of sample thickness for static and transient XAS at the N K-edge in fluorescence detection geometry. (b) SNR as a function of incident photon counts. The dashed line indicates SNR = 10.

To enable a consistent comparison between the static and transient cases, the SNR was optimised numerically by minimising −SNR(*X*) over the relevant thickness range. The optimal thickness was defined as the smallest *X* for which the SNR reached 99.9% of its maximum value. This criterion avoids selecting unnecessarily thick films once the SNR has saturated, while preserving the optimum in the transient case. The resulting optimal thickness for the N K-edge for the transient case is approximately 79 nm, in contrast to the static case, where the SNR continues to increase up to thicknesses approaching 1 µm.

The obtained optimal thickness was subsequently used to evaluate the SNR as a function of incident photon count, as shown in Fig. 2(b). The transient regime requires significantly higher photon counts to achieve a given SNR (*e.g.* SNR = 10), reflecting both the reduced excitation fraction and the additional 
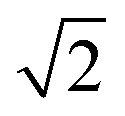
 noise factor associated with pump–probe measurements. For the N K-edge, achieving an SNR of 10 requires approximately two orders of magnitude more photons in the transient case compared to the static case, highlighting the stringent flux requirements for time-resolved measurements at soft X-ray edges in weakly absorbing systems.

The SNR model was applied to all relevant absorption edges within the water window, and the results are summarised in Table 1.

**Table 1:** Comparison of optimal sample thickness, required incident photon counts, and estimated acquisition time to achieve SNR = 10 for static and transient XAS measurements of Y6 in fluorescence detection geometry (*ψ* = 90°, *ϕ* = 45°). The acquisition time assumes an incident photon flux of ∼2 × 10^13^ photons s^−1^, corresponding to ∼10^10^ photons per pulse at 5.5 kHz attenuated with a 780 nm Al filter

Edge	Static			Transient^a^		
	*L*_opt_ (µm)	*I*_0_ (counts)	Acquisition time (s)	*L*_opt_ (µm)	*I*_0_ (counts)	Acquisition time (s)
S L_2,3_	0.9825	3.66 × 10^11^	0.018	0.0883	1.15 × 10^14^	5.75
C K	0.6748	6.29 × 10^10^	0.003	0.0838	1.46 × 10^13^	0.73
N K	0.9080	1.05 × 10^12^	0.052	0.0791	2.21 × 10^14^	11.0
O K	0.9853	2.56 × 10^12^	0.128	0.0893	8.46 × 10^14^	42.3
F K	0.9943	1.13 × 10^12^	0.057	0.0966	5.42 × 10^14^	27.1

a Pump wavelength = 800 nm; fluence = 300 µJ cm^−2^; Y6 *µ*_*λ*_ = 10.9 µm^−1^

Using an expected incident photon flux of ∼2 × 10^13^ photons s^−1^ at the chemRIXS endstation (for 5.5 kHz repetition rate, 780 nm Al attenuation, and ∼4 J pulse energy at the interaction point for 400 eV photons^21^), we estimate the acquisition time required to reach SNR = 10 for both static and transient measurements.

For static XAS, the required photon counts are modest, corresponding to acquisition times in the millisecond to sub-second range across all edges. These conditions are well within experimentally accessible limits and can be achieved before significant radiation damage occurs.

In contrast, transient measurements require substantially higher photon counts due to the additional scaling with excitation fraction, leading to acquisition times increased by several orders of magnitude. For heavier edges (N, O, F), this corresponds to acquisition times of ∼10–40 seconds. The photon counts required to reach SNR = 10 in this regime approach the maximum tolerable dose that preserves the optical response of Y6 (≲2 × 10^16^ photons cm^−2^).

This establishes a practical constraint: the achievable SNR in transient measurements is not solely limited by photon statistics but by the allowable dose per sample region. Consequently, experimental parameters such as beam size and raster scanning speed must be carefully chosen to distribute the dose while maintaining sufficient signal levels.

These results highlight that, while static TFY-XAS is readily feasible, transient measurements are fundamentally constrained by radiation damage. Optimising experimental conditions therefore requires operating within a dose-limited regime, balancing sample thickness, excitation fraction, and incident flux to maximise signal while preserving the sample response.

Although the SNR model defines these fundamental limits, the reliable extraction of XAS spectra from shot-resolved data additionally depends on the statistical properties of the measured signals and the analysis methodology employed. We therefore now turn to the evaluation of data-driven approaches for extracting XAS from experimentally measured TFY signals.

### 5.2 Results on the extraction of XAS from measured TFY data

The precision and stability of the extracted spectra depend not only on the achievable SNR, but also on the statistical relationship between the fluorescence signal *I*_f,*j*_ and the incident flux estimate *I*_0,*j*_. To quantify this relationship, we use Pearson’s correlation coefficient *r*.^67^

In the following, we evaluate two related questions: first, whether a calibration-based regression model can improve the incident-flux proxy used for normalisation; and second, whether the XAS observable is more reproducibly extracted using conventional ratio-based estimators or shot-resolved fit-based estimators.

The analysis is performed on two datasets acquired under nominally identical conditions using 60 nm thick Y6 samples, with the incident photon flux varied using aluminium attenuation. The low-dose regime (780 nm filter; runs 54, 53, 48, 47, and 46) lies within the dose limits required for transient measurements, where preservation of the optical response is essential but the fluorescence signal and *I*_0_–*I*_f_ correlation are weak. The intermediate-dose regime (400 nm filter; runs 151, 130, 45, 44, and 43) provides improved signal levels and correlation, and is therefore more representative of static XAS conditions, although it approaches the onset of radiation-induced effects.

#### 5.2.1 Training of the regression model for flux reconstruction

We first assess whether the regression model can be trained directly on the target dataset. Using Y6 data, the model was trained to predict the fluorescence signal *I*^(pred,Y6)^_f,*j*_ from the measured detector vector *x̂*_*j*_, and subsequently evaluated across all Y6 runs. In this case, only marginal improvement in correlation was observed, with Pearson correlation coefficients remaining below *r* ∼ 0.5.

To test whether this limitation arises from temporal variations in the experiment, the training was restricted to temporally local subsets (*e.g.* ∼10^3^ shots at fixed sample position). This approach aims to minimise the influence of slow drifts, such as those induced by sample rastering or beam pointing instabilities. However, no significant improvement in performance was observed. This indicates that the limitation is not primarily due to temporal non-stationarity, but rather reflects the intrinsically low fluorescence yield under dose-limited conditions, where shot noise dominates the signal.

In contrast, measurements on the reference sample (hBN) exhibit a substantially stronger correlation (*r* ∼ 0.9) between the measured fluorescence signal *I*^(ref)^_f,*j*_ and the incident flux *I*^(ref)^_0,*j*_. As shown in Fig. 3(a), the regression model further improves this correlation, yielding *r* > 0.95 across the energy range.

**Fig. 3:**
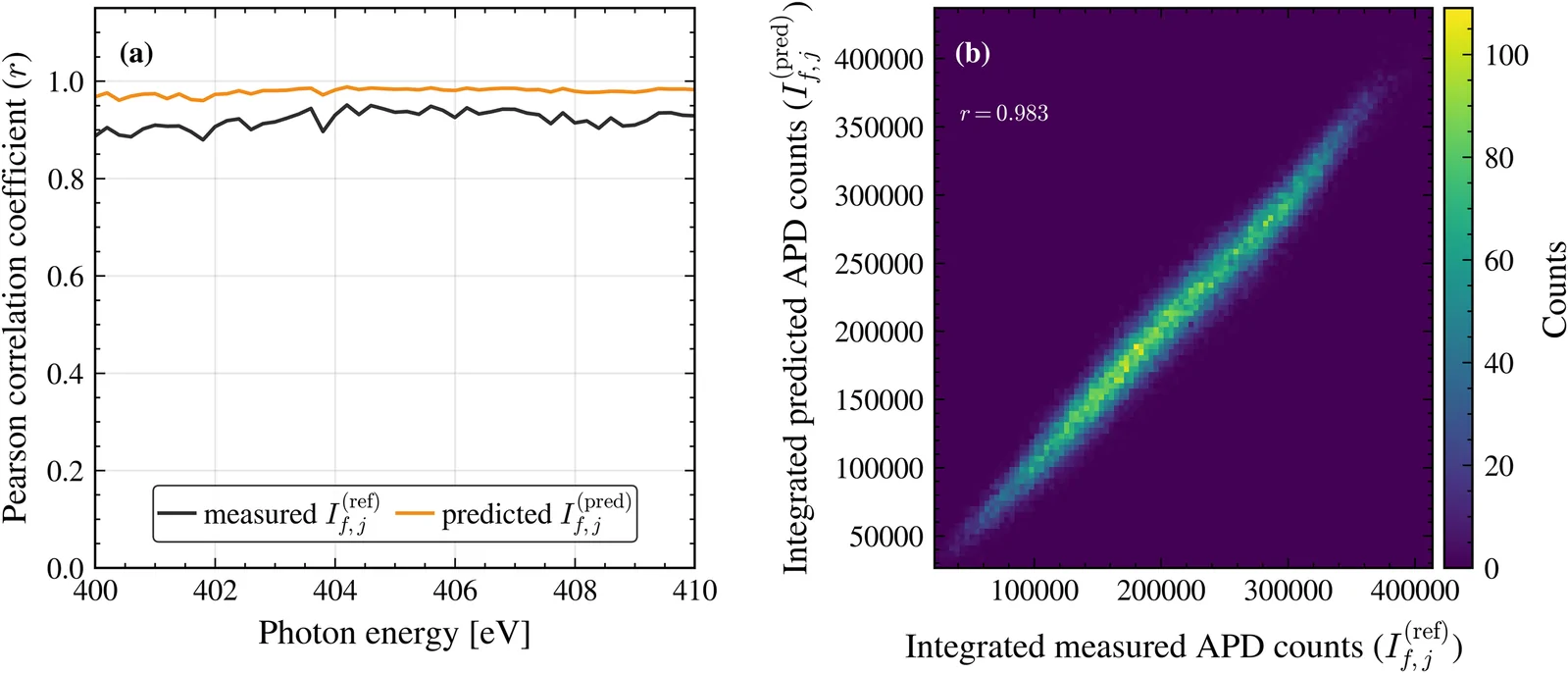
Calibration of the regression model using the hBN reference dataset. (a) Pearson correlation coefficient *r* between the incident flux signal *I*^(ref)^_0,*j*_ and the fluorescence signal for both the raw measured signal *I*^(ref)^_f,*j*_ (black) and the regression-based prediction *I*^(pred)^_f,*j*_ (orange) as a function of photon energy. (b) Comparison of predicted and measured fluorescence signals at a representative photon energy (405 eV), showing strong linear agreement (*r* = 0.983).

Fig. 3(b) shows *I*^(pred)^_f,*j*_ plotted against *I*^(ref)^_f,*j*_ at a representative photon energy (405 eV). The strong linear relationship (*r* = 0.983) demonstrates that the model accurately captures the mapping between detector signals and fluorescence response. Deviations from linearity are minimal, indicating that the regression effectively accounts for detector non-idealities such as saturation and channel-dependent response variations.

These results suggest that the detector response in the hBN measurements is sufficiently well conditioned to enable reliable calibration of the mapping between incident flux signals and fluorescence. We therefore train the regression model on the hBN dataset and apply the resulting coefficients to the Y6 measurements to construct a shot-resolved incident flux proxy, *I*^(pred)^_0,*j*_, as defined in eqn (32).

#### 5.2.2 Application of the calibrated flux proxy to Y6 measurements

We now evaluate the performance of the calibrated regression model when applied to the Y6 dataset, focusing on its ability to improve the correlation between the fluorescence signal *I*_f,*j*_ and the incident flux estimate.

Fig. 4(a and b) show the correlation coefficient *r*(*E*) as a function of photon energy for the measured and regression-derived flux signals, respectively. In all cases, the use of the regression-based proxy leads to a systematic increase in correlation across the energy range. The improvement is most pronounced near the absorption edge, where fluctuations in signal intensity are largest.

**Fig. 4:**
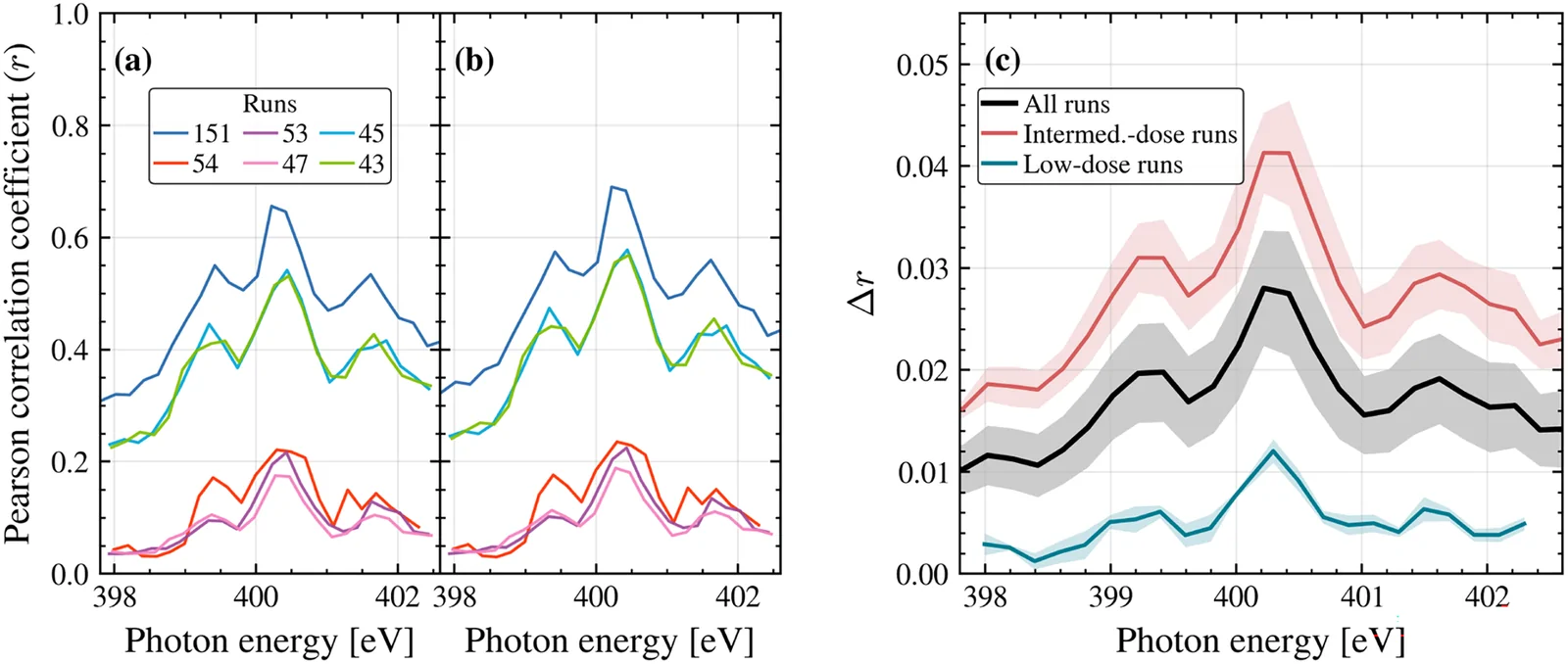
(a) Pearson correlation coefficient *r*(*E*) between *I*_f,*j*_ and the measured incident flux *I*_0,*j*_ as a function of photon energy for representative runs spanning both low-dose (*N*_*γ*_ ≲ 2 × 10^16^ photons cm^−2^; runs 54, 53, 47) and intermediate-dose (5 × 10^16^ ≲ *N*_*γ*_ ≲ 10^17^ photons cm^−2^; runs 151, 45, 43) regimes. (b) Same as (a), but using the regression-derived flux proxy *I*^(pred)^_0,*j*_. (c) Mean correlation improvement 〈Δ*r*(*E*)〉 = 〈*r*_pred_(*E*) − *r*_measured_(*E*)〉 for all runs (black), the intermediate-dose regime (magenta), and the low-dose regime (teal). Shaded regions indicate the standard deviation across runs.

The gain in correlation is quantified in Fig. 4(c), which shows the mean difference

35 〈Δ*r*(*E*)〉 = 〈*r*_pred_(*E*) − *r*_measured_(*E*)〉,

evaluated separately for the intermediate-dose regime runs, the low-dose regime runs, and the combined dataset.

To compare the overall improvement between regimes, we further define the energy-averaged correlation improvement36
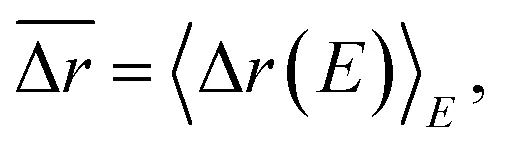
evaluated over the photon-energy window 398 ≤ *E* ≤ 402.5 eV.

Across all runs, the regression-derived flux proxy increases the correlation by 
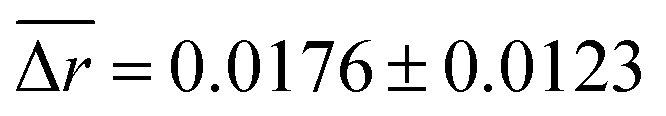
, corresponding to a mean relative improvement of 6.65 ± 2.95%.

The magnitude of the improvement depends strongly on the accumulated dose regime. For the intermediate-dose regime (5 × 10^16^ ≲ *N*_*γ*_ ≲ 10^17^ photons cm^−2^; runs 151, 130, 45, 44, and 43), the average improvement is 
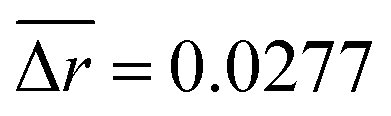
, corresponding to a relative increase of 7.82%. In contrast, for the low-dose, dose-limited regime (*N*_*γ*_ ≲ 2 × 10^16^ photons cm^−2^; runs 54, 53, 47, and 48), the improvement is substantially smaller, 
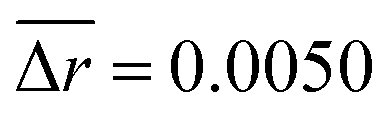
, corresponding to a relative increase of 5.17%.

This behaviour reflects the underlying signal statistics. In the intermediate-dose regime, where the fluorescence signal is stronger, the regression model can more effectively refine the incident-flux proxy and improve the correlation between *I*_0,*j*_ and *I*_f,*j*_. In the low-dose regime, the weak fluorescence signal intrinsically limits the achievable correlation, and consequently limits the absolute benefit of flux reconstruction.

While systematic, the improvement obtained from the regression-derived flux proxy alone remains modest, particularly in the low-dose regime most relevant for transient measurements. This suggests that improving the incident-flux estimate alone is insufficient to fully stabilise the extracted XAS spectra under dose-limited conditions.

We therefore next examine the influence of the XAS estimator itself, comparing ratio-based and regression-based approaches using both the measured and regression-derived flux signals.

#### 5.2.3 Comparison of XAS estimators

We now compare the extraction of XAS from shot-resolved fluorescence data using two estimators: the ratio-of-means (ROM), eqn (22), and the ordinary least-squares (OLS) fit-slope estimator, eqn (26). Each is evaluated using both the measured incident flux *I*_0,*j*_ and the regression-derived proxy *I*^(pred)^_0,*j*_.

All spectra were baseline-subtracted using the pre-edge region (*E* < 398.5 eV) and normalised to unit area over 398.5 ≤ *E* ≤ 402.5 eV to ensure that differences reflect spectral shape and reproducibility.

Run-to-run variability is quantified *via* the standard deviation37
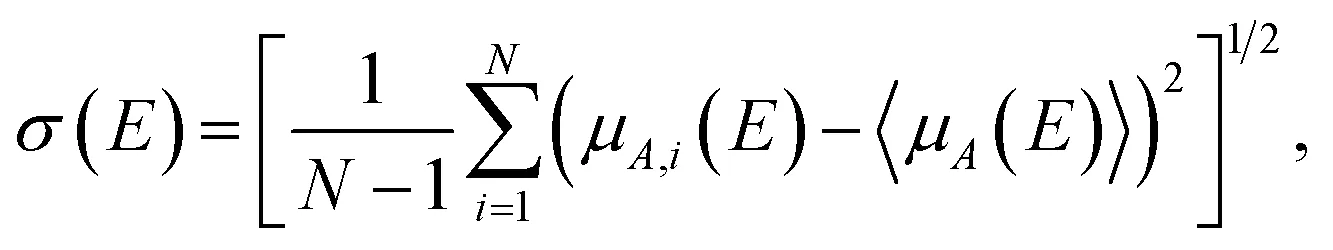
and its energy-averaged value

38 *σ̄* = 〈*σ*(*E*)〉_*E*_,

evaluated over 398 ≤ *E* ≤ 402.5 eV.

Fig. 5 compares the extracted XAS spectra obtained using the different estimators. Significant variability is observed for the ROM approach when using the measured incident flux *I*_0,*j*_ (Fig. 5(a)), with several runs exhibiting pronounced distortions and non-physical spectral features. The overall run-to-run variability is largest for this method, with *σ̄* = 0.0710.

**Fig. 5:**
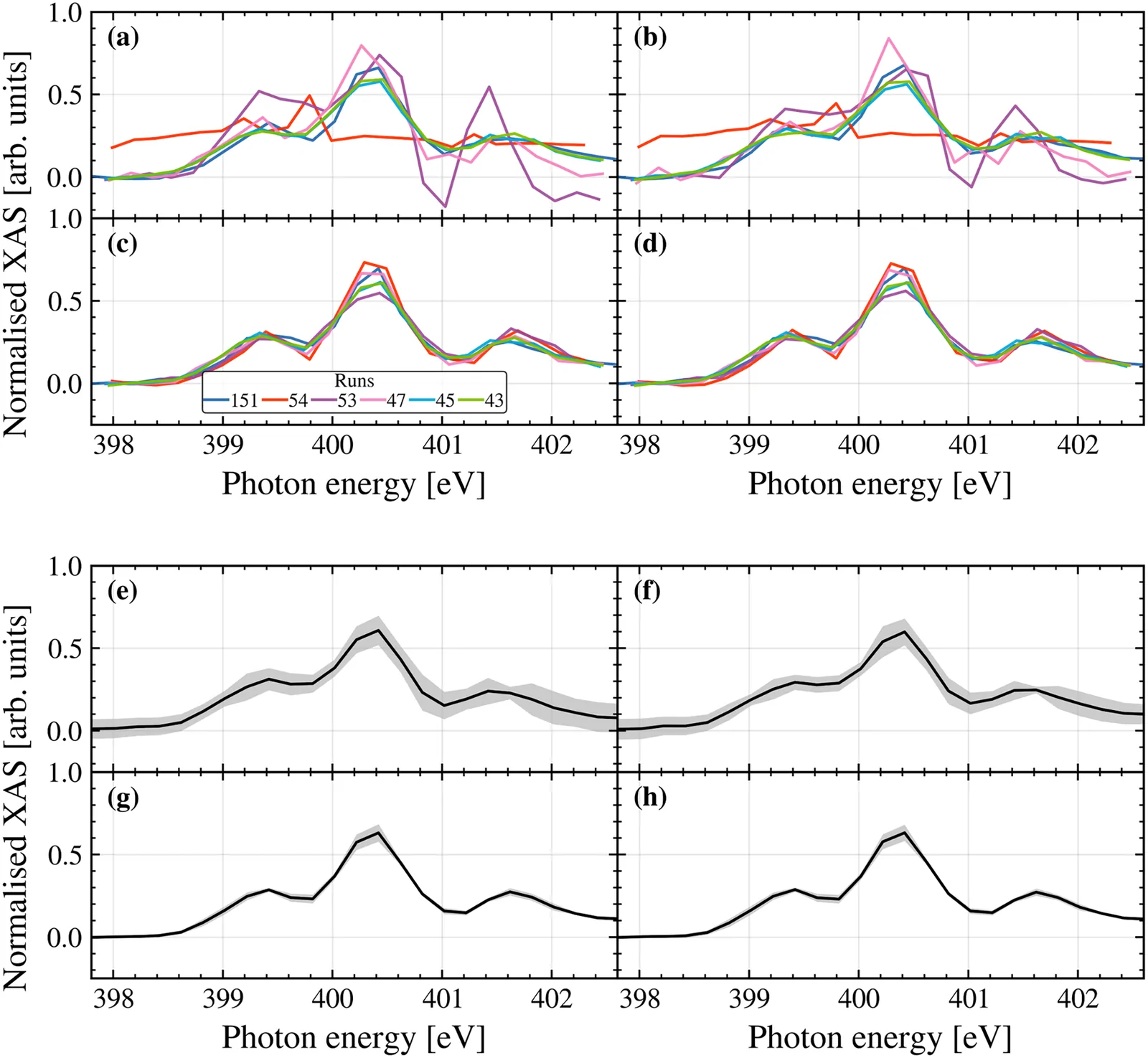
Comparison of XAS extraction methods across two dose regimes. (a–d) Normalised XAS spectra for representative runs acquired in the low-dose regime (*N*_*γ*_ ≲ 2 × 10^16^ photons cm^−2^; runs 54, 53, 47) and the intermediate-dose regime (5 × 10^16^ ≲ *N*_*γ*_ ≲ 10^17^ photons cm^−2^; runs 151, 45, 43). (a) Ratio-of-means using measured incident flux *I*_0,*j*_. (b) Ratio-of-means using regression-derived flux proxy *I*^(pred)^_0,*j*_. (c) OLS fit-slope estimator using measured *I*_0,*j*_. (d) OLS fit-slope estimator using *I*^(pred)^_0,*j*_. (e–h) Corresponding mean spectra over all runs, with shaded regions indicating the run-to-run standard deviation *σ*(*E*).

This behaviour is strongly dependent on the accumulated dose regime. In the intermediate-dose regime, the ROM estimator yields a comparatively moderate variability of *σ̄* = 0.0257. In contrast, the low-dose regime exhibits substantially larger variability, *σ̄* = 0.0859, reflecting the weak shot-to-shot correlation between *I*_0,*j*_ and *I*_f,*j*_ under dose-limited conditions where the fluorescence signal approaches the detector noise floor.

Replacing the measured incident flux with the regression-derived proxy *I*^(pred)^_0,*j*_ (Fig. 5(b)) leads to a systematic but modest improvement in spectral consistency. The overall variability decreases from *σ̄* = 0.0710 to 0.0589, corresponding to a reduction of approximately 17%. The improvement is somewhat larger in the intermediate-dose regime, where *σ̄* decreases from 0.0257 to 0.0210, than in the low-dose regime, where it decreases from 0.0859 to 0.0762. Substantial run-to-run variability therefore remains, particularly for the low-dose datasets with the weakest *I*_0,*j*_–*I*_f,*j*_ correlations. This indicates that improving the incident-flux estimate alone is insufficient to stabilise the extracted XAS under dose-limited conditions.

A substantially larger improvement is obtained when the XAS is extracted using the OLS fit-slope estimator (Fig. 5(c and d)). In this case, the spectra become significantly more reproducible across runs, even when using the measured incident flux signal. Using measured *I*_0,*j*_, the overall variability decreases to *σ̄* = 0.0195, while the combination of the OLS estimator with the regression-derived proxy *I*^(pred)^_0,*j*_ yields the lowest variability, *σ̄* = 0.0193.

The improvement obtained using the OLS estimator is most pronounced in the low-dose regime. Here, the variability decreases from *σ̄* = 0.0859 for the ROM estimator using measured *I*_0,*j*_ to *σ̄* = 0.0196 for the OLS estimator using *I*^(pred)^_0,*j*_, corresponding to a reduction by a factor of approximately 4.4. In the intermediate-dose regime, the corresponding reduction is more modest, from 0.0257 to 0.0136, or approximately a factor of 1.9. The largest gains therefore occur precisely in the regime most strongly constrained by radiation damage and most relevant for transient measurements.

These results indicate that the dominant improvement in XAS reproducibility arises from the choice of estimator rather than from the quality of the incident-flux estimate itself. Reformulating the extraction as a shot-resolved regression problem substantially suppresses the sensitivity to baseline offsets and statistical fluctuations, enabling stable and physically meaningful XAS spectra to be recovered even in the dose-limited regime.

To identify the origin of the discrepancy between the ROM and OLS estimators, we examine the shot-resolved (*I*^(pred)^_0,*j*_,*I*_f,*j*_) distribution at a photon energy near the absorption maximum (400.5 eV) for Run 54, a representative dataset from the low-dose regime.

The conditional variance Var(*I*_f_∣*I*_0_) grows monotonically with the conditional mean and shows no additive noise floor (Fig. 6(b)), consistent with shot-noise-dominated detection of the fluorescence. The clearest deviation from the Poisson–Poisson model is therefore not in the noise structure but in the mean structure: the linear fit to the conditional mean extrapolates to a non-zero intercept *â*_OLS_ corresponding to ∼40% of the average fluorescence signal at this photon energy. This intercept reflects an integrated detector baseline rather than the absorption signal. Because ROM enforces the through-origin assumption, this offset is absorbed into the slope. From eqn (22) and (26), the two estimators are related by the exact identity39
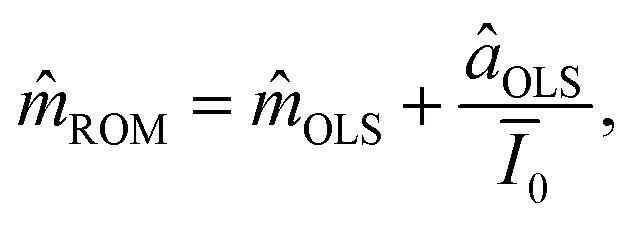
which is consistent with our data.

**Fig. 6:**
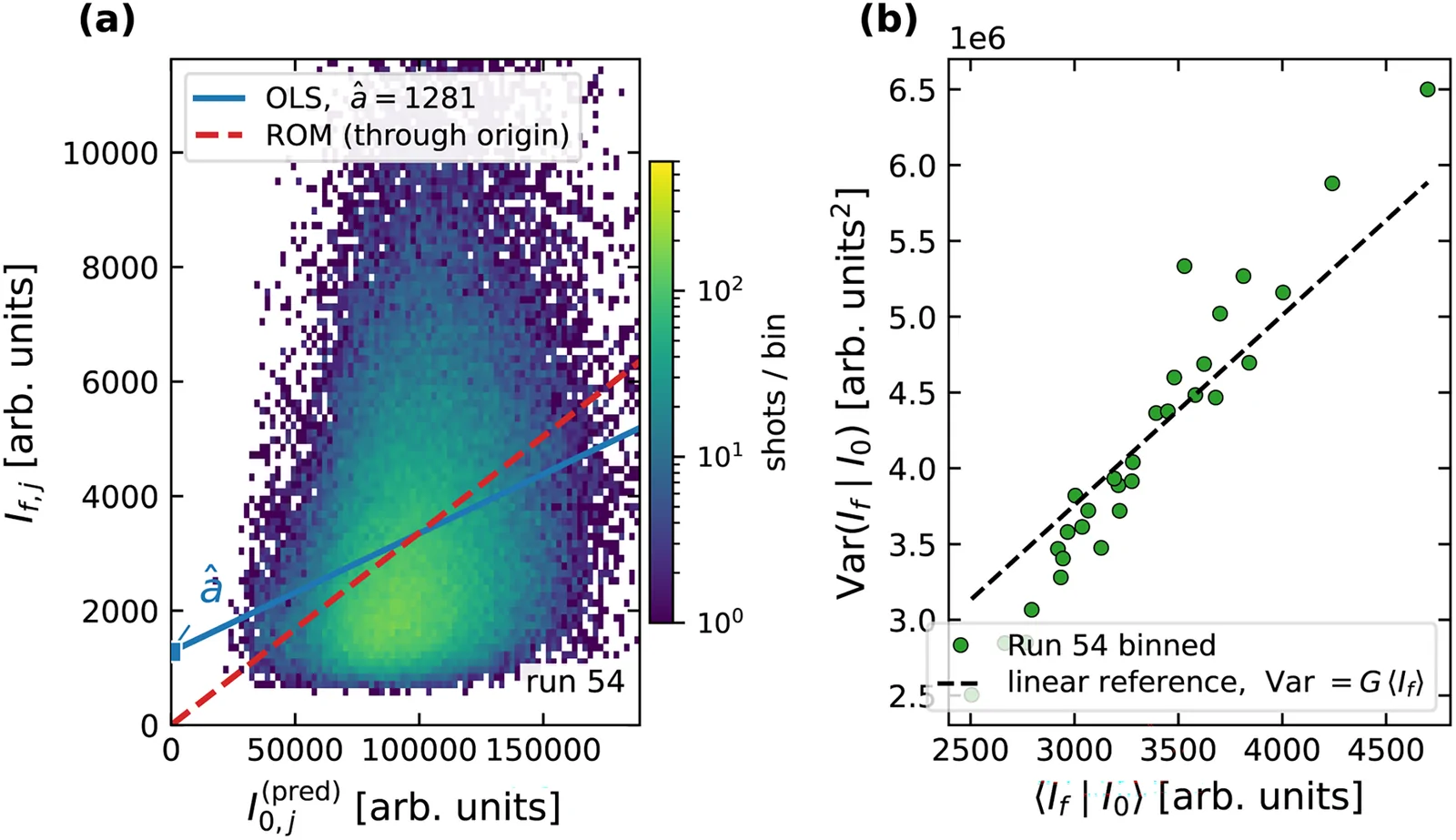
Distribution of *I*_f_ given *I*_0_ in Run 54 at 400.5 eV. (a) 2D histogram of fluorescence *I*_f,*j*_*versus* predicted incident flux *I*^(pred)^_0,*j*_. The OLS fit (solid blue) recovers a non-zero intercept *â*_OLS_ (square marker). The ROM fit (dashed red), forced through the origin, absorbs this pedestal into the slope. (b) Conditional variance Var(*I*_f_∣*I*_0_) as a function of conditional mean 〈*I*_f_∣*I*_0_〉, computed in *I*_0_ quantile bins. The dashed line is a through-origin reference; the variance grows monotonically with the mean.

The pedestal *â*_OLS_ is approximately stationary, but the average incident flux *Ī*_0_ varies between runs (sample drift, beam pointing) and across the absorption edge through the monochromator transmission. The bias term *â*_OLS_/*Ī*_0_ therefore imprints a run- and energy-dependent additive distortion on the extracted spectrum, which propagates directly into the run-to-run variance of Table 2. By including an intercept, the OLS estimator effectively performs a dynamic background subtraction at every photon energy, decoupling the absorption signal from the detector baseline.

**Table 2:** Comparison of XAS extraction methods. Values correspond to the energy-averaged run-to-run standard deviation *σ̄* over 398 ≤ *E* ≤ 402.5 eV. Results are shown separately for the intermediate-dose regime (5 × 10^16^ ≲ *N*_*γ*_ ≲ 10^17^ photons cm^−2^; runs 151, 130, 45, 44, 43), the low-dose regime (*N*_*γ*_ ≲ 2 × 10^16^ photons cm^−2^; runs 54, 53, 48, 47), and the combined dataset

Extraction method	Intermediate-dose	Low-dose	All runs
ROM, *I*_0,*j*_	0.0257	0.0859	0.0710
ROM, *I*^(pred)^_0,*j*_	0.0210	0.0762	0.0589
OLS, *I*_0,*j*_	0.0136	0.0197	0.0195
OLS, *I*^(pred)^_0,*j*_	0.0136	0.0196	**0.0193**

#### 5.2.4 Outlier rejection

To assess whether the OLS estimator is sensitive to outliers, we apply a residual-based rejection scheme using the median absolute deviation (MAD).^68^ This tests whether the tails of the shot-to-shot *I*_0,*j*_–*I*_f,*j*_ distribution arise from detector artefacts or instead reflect intrinsic XFEL and fluorescence fluctuations.

Starting from the global OLS fit,

40 *I*_f,*j*_ = *m̂*_OLS_*I*_0,*j*_ + *â*_OLS_,

we define the residuals

41 *ε*_*j*_ = *I*_f,*j*_ − (*m̂*_OLS_*I*_0,*j*_ + *â*_OLS_).

As discussed in the previous section, the conditional variance Var(*I*_f_∣*I*_0_) increases with intensity (Fig. 6(b)), indicating a heteroscedastic shot distribution. To avoid preferential rejection of high-intensity shots, the residual spread is estimated locally as a function of *I*_0,*j*_.

Shots are sorted by *I*_0,*j*_ and divided into quantile bins containing approximately equal numbers of shots. Within each bin *q*, we compute the local MAD,

42 MAD_*q*_ = median_*j*∈*q*_(|*ε*_*j*_ − median_*j*∈*q*_(*ε*_*j*_)|),

and convert it to a Gaussian-equivalent scale,

43 *σ*_MAD,*q*_ = 1.4826 × MAD_*q*_.

Shots in bin *q* are retained if

44 |*ε*_*j*_ − median_*j*∈*q*_(*ε*_*j*_)| < *kσ*_MAD,*q*_,

after which the OLS fit is recomputed using only the retained subset of shots, *N*_*k*_.

Rather than reporting *k* directly, we parameterise the clipping strength using the mean retained shot fraction 〈*N*_*k*_/*N*〉, averaged over the energy window 398 ≤ *E* ≤ 402.5 eV and over all runs. In the following discussion, aggressive rejection corresponds to *k* = 2 (〈*N*_*k*_/*N*〉 ≈ 0.90), moderate rejection to *k* = 3–3.5 (〈*N*_*k*_/*N*〉 ≈ 0.95–0.97), and weak rejection to *k* ≳ 4 (〈*N*_*k*_/*N*〉 ≳ 0.97).

Fig. 7 shows the energy-averaged variability *σ̄* as a function of retained shot fraction for both measured and regression-derived incident flux estimates. In all cases, the minimum variability is obtained for the global fit without rejection (〈*N*_*k*_/*N*〉 = 1). As progressively more shots are removed, the extracted XAS becomes systematically less reproducible.

**Fig. 7:**
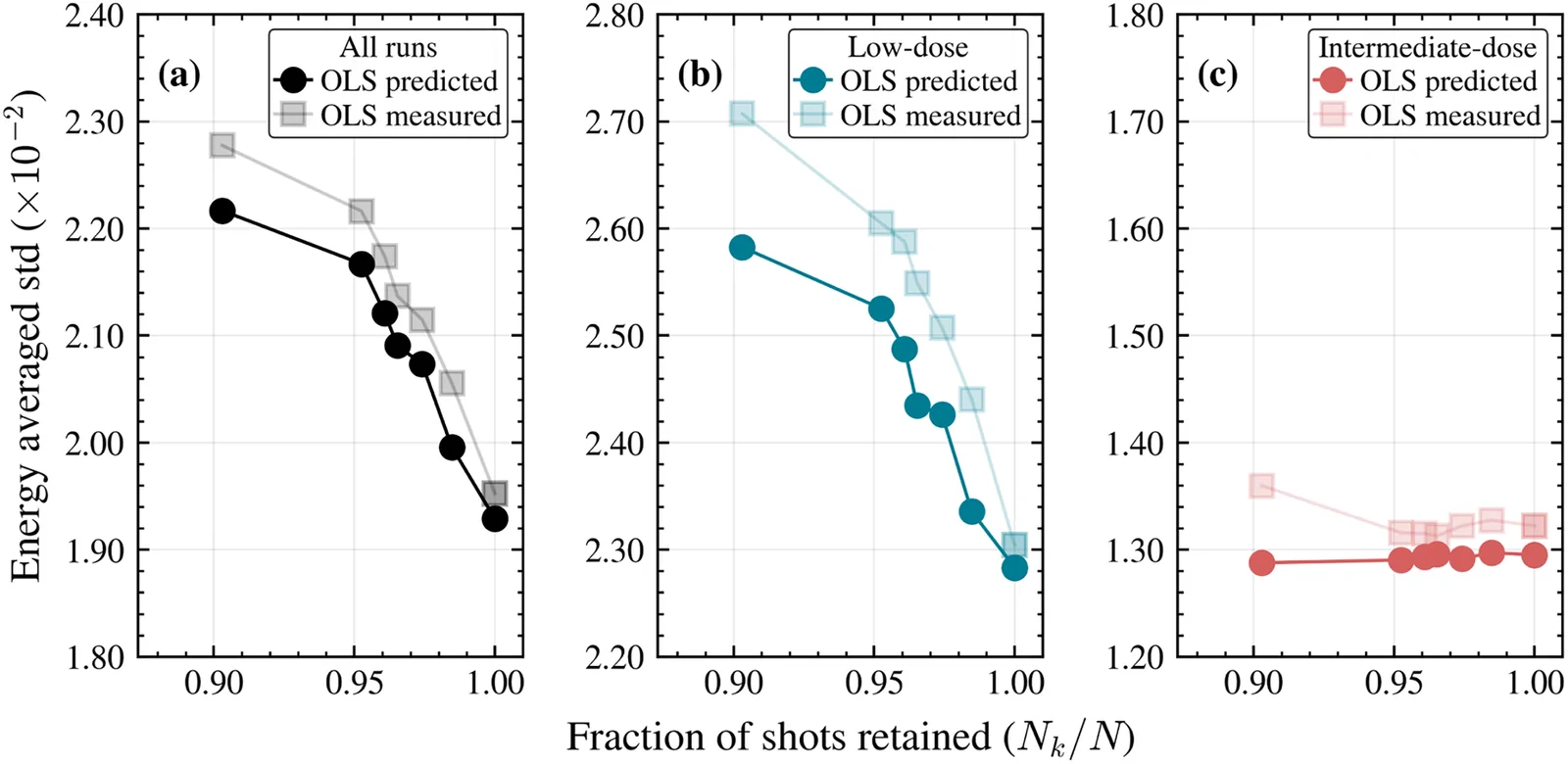
Effect of residual-based outlier rejection on the stability of the OLS estimator. Energy-averaged standard deviation *σ̄* of the extracted XAS as a function of the mean retained shot fraction 〈*N*_*k*_/*N*〉 after local MAD-based residual rejection. Results are shown separately for (a) the combined dataset, (b) the low-dose regime (*N*_*γ*_ ≲ 2 × 10^16^ photons cm^−2^), and (c) the intermediate-dose regime (5 × 10^16^ ≲ *N*_*γ*_ ≲ 10^17^ photons cm^−2^). Filled circles correspond to the OLS estimator using the regression-derived flux proxy *I*^(pred)^_0,*j*_, while open squares show the corresponding results using the measured incident flux *I*_0,*j*_. Increasingly aggressive rejection (smaller 〈*N*_*k*_/*N*〉) systematically increases the variability of the extracted XAS, particularly in the low-dose regime.

For the combined dataset, using the regression-derived proxy *I*^(pred)^_0,*j*_, the variability increases from *σ̄* = 0.0193 without rejection to ∼0.021 under moderate rejection and to ∼0.022 under aggressive rejection. A similar trend is observed using the measured incident flux *I*_0,*j*_.

The effect is strongest in the low-dose regime, where the fluorescence signal is weak and shot noise dominates the statistics. Here, *σ̄* increases from 0.0228 without rejection to ∼0.026–0.027 under aggressive rejection. In contrast, the intermediate-dose regime is comparatively insensitive to rejection, with *σ̄* remaining close to ∼0.013 across the full rejection range.

These results indicate that the apparent outliers in the *I*_0,*j*_–*I*_f,*j*_ distribution are not dominated by detector artefacts, but instead correspond largely to physically meaningful shot-to-shot fluctuations. Removing these points reduces the effective sample size and degrades the statistical precision of the fitted slope.

Importantly, the degradation is strongest in the low-dose regime most relevant for transient measurements. Under these dose-limited conditions, retaining the full shot distribution is essential for maximising statistical efficiency.

We therefore conclude that the optimal strategy is to use a global OLS fit between *I*_f,*j*_ and *I*^(pred)^_0,*j*_ without explicit outlier rejection. This approach maximises reproducibility while preserving the intrinsic statistical fluctuations present in the XFEL data.

Having established the optimal statistical framework for extracting XAS, we now examine how experimental parameters influence the underlying signal quality. In particular, we focus on the role of sample thickness, which directly affects fluorescence yield and therefore the correlation between *I*_0,*j*_ and *I*_f,*j*_.

#### 5.2.5 Effect of sample thickness on fluorescence signal and correlation

We next examine the effect of sample thickness on the fluorescence signal and its correlation with the incident flux. The measurements presented in this section were acquired in a different detection geometry from the previous datasets, with *ψ* = 90° and *ϕ* = 45°, and were performed in the intermediate-dose regime (5 × 10^16^ ≲ *N*_*γ*_ ≲ 10^17^ photons cm^−2^).

Fig. 8 shows the shot-to-shot distributions of fluorescence *versus* incident flux for two representative film thicknesses (60 nm and 100 nm), together with the corresponding Pearson correlation coefficient as a function of photon energy.

**Fig. 8:**
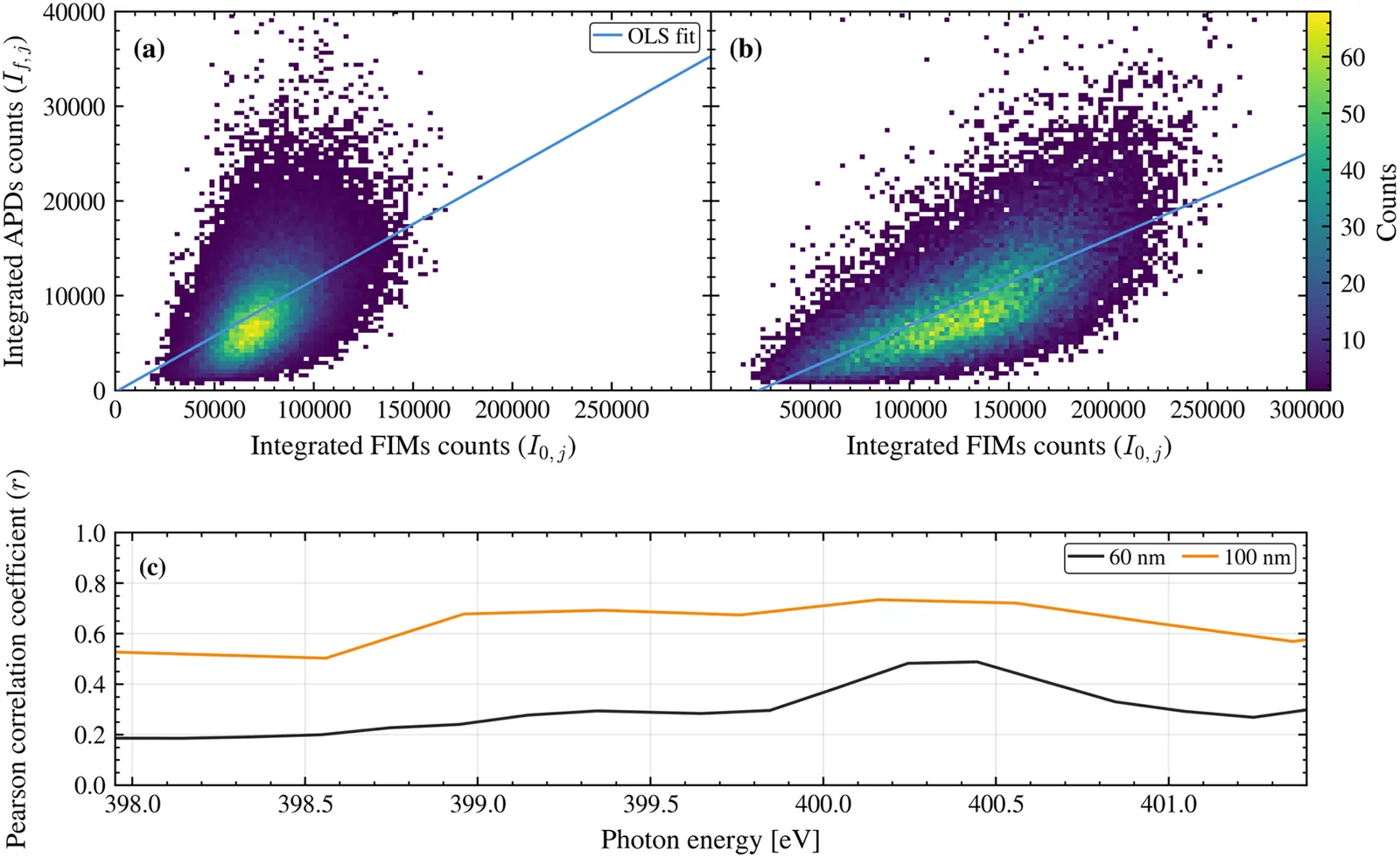
Effect of sample thickness on fluorescence signal and correlation. Measurements were acquired in the intermediate-dose regime (5 × 10^16^ ≲ *N*_*γ*_ ≲ 10^17^ photons cm^−2^) using a detection geometry of *ψ* = 90° and *ϕ* = 45°. (a and b) Shot-to-shot distributions of fluorescence (*I*_f,*j*_) *versus* incident flux (*I*_0,*j*_) for (a) 60 nm and (b) 100 nm films, respectively. (c) Pearson correlation coefficient as a function of photon energy for both thicknesses. The thicker sample exhibits higher fluorescence yield and improved correlation.

The thicker sample exhibits a systematically higher fluorescence signal, leading to a tighter clustering of points in the *I*_0,*j*_–*I*_f,*j*_ plane and an increased correlation coefficient across the energy range. This improvement is most pronounced near the absorption edge, where the fluorescence yield is largest. Averaged over energy, the correlation is significantly enhanced for the thicker film, reflecting improved counting statistics and reduced relative shot noise.

This behaviour is consistent with the SNR model introduced in Section 3, where increasing sample thickness enhances the detected fluorescence signal and reduces the relative contribution of shot noise prior to saturation.

However, this trend does not directly translate to time-resolved measurements. In pump–probe experiments, increasing the sample thickness reduces the fraction of photoexcited molecules probed by the X-ray pulse due to the mismatch between optical and X-ray absorption depths. As a result, while thicker samples improve the static SNR, they can suppress the transient signal by reducing the effective excitation fraction.

The experimental observations are therefore consistent with the predicted trade-off: thicker samples (within the thin-sample regime *X* ≪ 0.5 µm) provide improved signal stability and correlation in static measurements, but may become suboptimal in the transient regime where sensitivity to pump-induced changes is required. This highlights the importance of tailoring the sample thickness to the specific measurement objective, balancing fluorescence signal strength against excitation fraction.

## 6 Summary and conclusions

In this work, we addressed a central challenge in XFEL-based soft X-ray spectroscopy: the reliable extraction of quantitative observables from shot-resolved measurements in low-signal, dose-limited regimes. While high-repetition-rate XFELs enable new classes of ultrafast measurements, the stochastic nature of the X-ray pulses, the limited fluorescence yield of organic thin films, and the radiation sensitivity of the sample place stringent constraints on both experimental design and data analysis.

The SNR model developed in Section 3 provides a quantitative framework for understanding these constraints. For fluorescence-detected XAS, the model predicts fundamentally different optimisation criteria for static and transient measurements. Static measurements benefit from increasing sample thickness until the fluorescence signal approaches saturation, whereas transient measurements require thinner films (∼80–100 nm) to maximise the fraction of photoexcited molecules probed by the X-ray pulse. This reflects the mismatch between optical and X-ray absorption depths. The model also shows that transient measurements require orders of magnitude higher photon counts than static measurements to reach the same SNR, bringing the required dose close to the radiation-damage limit of the sample.

Experimentally, we identified two relevant dose regimes. In the low-dose regime (*N*_*γ*_ ≲ 2 × 10^16^ photons cm^−2^), which is most relevant for transient measurements, no measurable radiation damage was observed, but the fluorescence signal and the *I*_0_–*I*_f_ correlation were weak. In the intermediate-dose regime (5 × 10^16^ ≲ *N*_*γ*_ ≲ 10^17^ photons cm^−2^), stronger fluorescence signals improved correlation and spectral stability, but signatures of optical bleaching began to appear. These regimes therefore define the central experimental trade-off between preserving sample response and obtaining sufficient fluorescence statistics.

A calibration-based flux reconstruction trained on hBN provided a systematic improvement in the correlation between the fluorescence signal and the incident-flux proxy. Across all runs, the regression-derived proxy increased the Pearson correlation coefficient by 
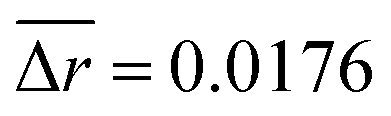
, corresponding to a mean relative improvement of 6.65%. The improvement was larger in the intermediate-dose regime (
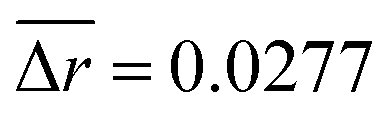
, 7.82%) than in the low-dose regime (
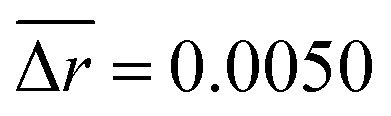
, 5.17%), indicating that flux reconstruction is most effective when the fluorescence signal is already sufficiently above the noise floor.

However, the dominant improvement in XAS reproducibility came from the choice of estimator. The conventional ratio-of-means estimator was highly sensitive to weak correlation and detector offsets, producing large run-to-run variability, particularly in the low-dose regime. Reformulating the observable as a shot-resolved OLS fit-slope estimator reduced the overall variability from *σ̄* = 0.0710 to 0.0193. The improvement was strongest in the low-dose regime, where *σ̄* decreased from 0.0859 to 0.0196, corresponding to a factor of ∼4.4 reduction. This demonstrates that stable and physically meaningful XAS spectra can be recovered even under dose-limited conditions when the observable is formulated as a regression problem.

The origin of this improvement was traced to a non-zero fluorescence pedestal in the shot-resolved *I*_0_–*I*_f_ distribution. Because the ratio-of-means estimator implicitly enforces a through-origin relationship, this pedestal is absorbed into the slope and produces run- and energy-dependent spectral distortions. In contrast, the OLS estimator includes an intercept and therefore dynamically separates the absorption-dependent signal from detector baseline contributions.

The optimal estimator for this data is independent of the photon energy. This is because the structure of noise in the detectors and the incident radiation is similar across all photon energies. This is expected to be the case for most XFEL experiments and if a large change in the relative performance of estimators across datasets acquired under comparable conditions is observed, the noise structure in those datasets should be investigated. If a significant change in the measurement configuration, for example the choice of detector or XFEL operation model, is made between measurements, there may be a case for evaluating the choice of estimator independently; however, this is very rarely the case.

We also tested residual-based outlier rejection using a local MAD procedure that accounts for the heteroscedastic shot distribution. Outlier rejection did not improve reproducibility. Instead, increasingly aggressive rejection reduced statistical efficiency and increased the variability of the extracted XAS, particularly in the low-dose regime. This indicates that the tails of the shot distribution primarily reflect intrinsic XFEL and fluorescence fluctuations rather than detector artefacts. Retaining the full shot distribution is therefore essential for maximising statistical precision.

Finally, measurements on films of different thickness confirmed the expected static-SNR behaviour. The thicker film produced stronger fluorescence signals and improved correlation with the incident flux, consistent with the SNR model. However, this benefit must be balanced against the reduced excitation fraction expected in pump–probe measurements, emphasising that the optimal thickness depends on whether the experiment targets static or transient observables.

Together, these results show that reliable XFEL observables require both physical modelling of the experiment and statistically appropriate inference of the measured signal. For dose-limited soft X-ray measurements on organic thin films, regression-based extraction provides a robust route to recovering XAS spectra in regimes where conventional TFY normalisation fails.

### 6.1 Future prospects

The approach developed here is not limited to Y6 or organic thin films. Similar challenges arise in liquid jets, dilute solutions, heterogeneous condensed-phase samples, and other low-yield systems where shot-to-shot flux fluctuations and weak fluorescence signals limit conventional normalisation. Calibration-based flux reconstruction and regression-based estimators may therefore provide a general route to recovering meaningful observables from noisy XFEL datasets.

We expect that the approach used here will be directly transferable to most systems studied with the same instrument. Larger differences may be expected between instruments and XFEL facilities, and continued collaboration within user communities and with facility scientists to understand the underlying sources of error at different instruments and how best to treat their data will help the community to make robust measurements of challenging systems.

Furthermore, these methods can be generalised to almost any XFEL measurement dependent on the correlation between two measured input observables. Correlating an observable with the incident flux is particularly common and our approach to improving the estimator for the incident flux would benefit many experiments. Statistical methods should develop alongside XFEL capabilities.

Future work should extend this framework to pump–probe data directly, where the additional constraints of excitation fraction, radiation damage, and pump-induced sample changes must be treated simultaneously. Incorporating nonlinear detector response, energy-resolved PFY/RIXS detection, and uncertainty estimation through resampling methods will further strengthen the connection between measured XFEL signals and the underlying molecular dynamics.

## Author contributions

B. A. G.: formal analysis, investigation, methodology, project administration, visualization, writing – original draft, writing – review and editing. M. M. M. N.: formal analysis, investigation, writing – original draft, writing – review and editing. O. G. A.: formal analysis, investigation, methodology, visualization, writing – original draft, writing – review and editing. K. H.: formal analysis, methodology, writing – review and editing. F. S. E.: formal analysis, investigation, writing – review and editing. Z. D.: formal analysis, investigation, writing – review and editing. J. J. B.: visualization, writing – review and editing. J. P. L.: visualization, writing – review and editing. D. J. H.: investigation, project administration, resources, writing – review and editing. K. K.: investigation, resources, writing – review and editing. M. B.: investigation, resources, writing – review and editing. D. G.: conceptualization, investigation, methodology, project administration, resources, supervision, writing – review and editing. O. G.: conceptualization, funding acquisition, project administration, supervision, writing – review and editing. M. R. M.: conceptualization, funding acquisition, supervision, writing – review and editing. J. P. M.: conceptualization, funding acquisition, project administration, supervision, writing – review and editing.

## Conflicts of interest

There are no conflicts to declare.

## Data Availability

Data supporting the conclusions of this study will be available from https://doi.org/10.5281/zenodo.22876087 following a priority access period for designated users. All other data needed to evaluate the conclusions in this paper are present in the paper.
